# Influence of Surface Roughness, Nanostructure, and Wetting on Bacterial Adhesion

**DOI:** 10.1021/acs.langmuir.3c00091

**Published:** 2023-04-04

**Authors:** Minchen Mu, Shuhao Liu, William DeFlorio, Li Hao, Xunhao Wang, Karla Solis Salazar, Matthew Taylor, Alejandro Castillo, Luis Cisneros-Zevallos, Jun Kyun Oh, Younjin Min, Mustafa Akbulut

**Affiliations:** † Artie McFerrin Department of Chemical Engineering, Texas A&M University, College Station, Texas 77843, United States; ‡ School of Chemistry and Chemical Engineering, Zhongkai University of Agriculture and Engineering, Guangzhou, Guangdong 510225, P. R. China; § Department of Chemical and Environmental Engineering, University of California, Riverside, California 92521, United States; ∥ Department of Food Science and Technology, Texas A&M University, College Station, Texas 77843, United States; ⊥ Department of Horticultural Sciences, Texas A&M University, College Station, Texas 77843, United States; ¶ Department of Polymer Science and Engineering, Dankook University, 152 Jukjeon-ro, Suji-gu, Yongin-si, Gyeonggi-do 16890, Republic of Korea

## Abstract

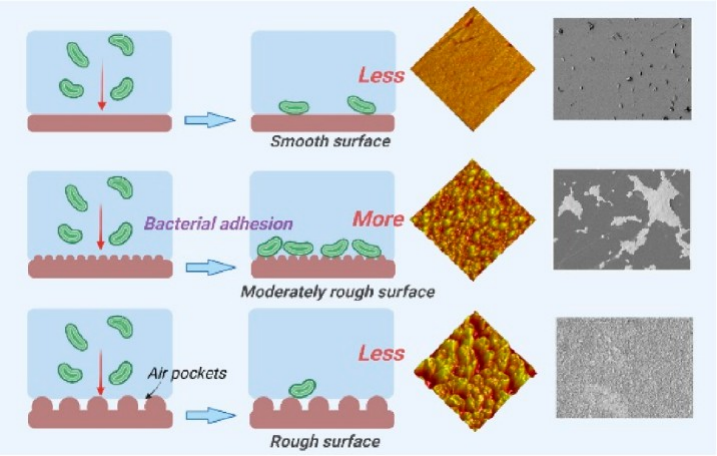

Bacterial fouling is a persistent problem causing the deterioration and failure of functional surfaces for industrial equipment/components; numerous human, animal, and plant infections/diseases; and energy waste due to the inefficiencies at internal and external geometries of transport systems. This work gains new insights into the effect of surface roughness on bacterial fouling by systematically studying bacterial adhesion on model hydrophobic (methyl-terminated) surfaces with roughness scales spanning from ∼2 nm to ∼390 nm. Additionally, a surface energy integration framework is developed to elucidate the role of surface roughness on the energetics of bacteria and substrate interactions. For a given bacteria type and surface chemistry; the extent of bacterial fouling was found to demonstrate up to a 75-fold variation with surface roughness. For the cases showing hydrophobic wetting behavior, both increased effective surface area with increasing roughness and decreased activation energy with increased surface roughness was concluded to enhance the extent of bacterial adhesion. For the cases of superhydrophobic surfaces, the combination of factors including (i) the surpassing of Laplace pressure force of interstitial air over bacterial adhesive force, (ii) the reduced effective substrate area for bacteria wall due to air gaps to have direct/solid contact, and (iii) the reduction of attractive van der Waals force that holds adhering bacteria on the substrate were summarized to weaken the bacterial adhesion. Overall, this study is significant in the context of designing antifouling coatings and systems as well as explaining variations in bacterial contamination and biofilm formation processes on functional surfaces.

## Introduction

For abiotic surfaces, bacterial adhesion (attachment) is a major problem leading to pathogenic contamination and/or performance losses of various devices, equipment, components, accessories, and vehicles used in healthcare, food and food packing, pipelines, and the maritime transportation industries.^1−3^ For biotic surfaces, bacterial adhesion is the first step in bacterial infections, vegetable and fruit spoilage, and plant diseases.^4−8^ At a colloidal level, bacterial adhesion to a surface corresponds to a transition from the planktonic state to the sessile state, involving several phases: (i) diffusive, convective, or flagellar motion of bacteria from bulk fluid until bacteria start to sense nanoscale intermolecular forces between bacteria and the surface, (ii) translocation of bacteria toward the surface due to the gradient of intermolecular interactions between bacteria and the surface along with the thermal energy, *k*_B_*T*, and flagellar kinetic energy (if present), (iii) approach of the bacterial cell wall or its appendages to the surface until the disjoining pressure of displacing the surface hydration layer is overcome, and (iv) molecular contact and anchoring between the substrate and bacterial wall/appendages.^9−15^

Prior studies have reported that there are several key properties and parameters of bacteria influencing bacterial adhesion to a surface, each to varying degrees. These include the hydrophobicity of the bacterial wall,^16^ surface potential of the bacteria,^17^ bacterial size,^18−20^ bacterial shape,^20−22^ the presence of curli and pili,^23−26^ quorum sensing ability,^27,28^ and the ability to produce extracellular bacterial adhesins.^29^ In addition, the characteristics of the substrate surface such as hydrophobicity,^30−32^ surface potential and charge,^33^ heterogeneity,^34,35^ patterns,^2,36,37^ roughness,^38−41^ and stiffness^42^ have been demonstrated to also be important. Some of the key external conditions governing bacterial adhesion trends are suspension pH,^43,44^ ionic strength,^45,46^ temperature,^47,48^ and the characteristics of any convective flow fields present.^49−52^

While there exist a large body of literature focusing on the influence of surface roughness on bacterial adhesion, the findings are conflicting. For instance, Yoda et al.^53^ investigated the effect of roughness on adhesion of *Staphylococcus epidermidis* to oxidized zirconium–niobium alloy (arithmetic mean surface roughness, or Ra, of 8.5 and 30.0 nm), cobalt–chromium–molybdenum alloy (Ra of 5.8 and 12.0 nm), titanium alloy (Ra of 7.1 and 16.5 nm), pure titanium (Ra of 5.6 and 22.0 nm), and stainless-steel surfaces (Ra of 1.8 and 7.2 nm). They found that there was increased bacterial adhesion on coarse surfaces compared to fine surfaces. Bohinc et al.^54^ prepared glass surfaces with five different roughnesses (0.07 μm, 0.58 μm, 0.99 μm, 2.5 μm, and 5.8 μm) and observed that the rate of *Escherichia coli (E. coli)*, *Staphylococcus aureus (S. aureus)*, and *Pseudomonas aeruginosa (P. aeruginosa)* adhesion increased with increasing surface roughness. However, Scheuerman et al.^55^ relied on silicon surfaces with 10, 20, 30, and 40 μm wide grooves of 10 μm depth (i.e., differing spacing parameters) to study the bacterial attachment behavior of *P. aeruginosa* and *Pseudomonas fluorescens*. They found that the rate of bacterial attachment was independent of groove size for all bacteria. Hilbert et al.^56^ reported that the adhesion of *Pseudomonas* sp., *Listeria monocytogenes*, and *Candida lipolytica* to stainless steel surfaces was not influenced by surface roughness in the Ra range of 0.01 to 0.9 μm. On the other hand, Truong et al.^57^ compared the adhesion of *P. aeruginosa* and *S. aureus* on titanium surfaces with root-mean-square (RMS) roughness of 223.3 and 84.2 nm. They observed that *S. aureus* and *P. aeruginosa* demonstrated preferential attachment to the smoother titanium surfaces that were prepared by equal channel angular pressing. Wu et al.^39^ reported that the number of adherent *P. aeruginosa* and *S. aureus* was significantly lower on rough stainless-steel surfaces as compared to the electropolished, smooth stainless-steel surfaces. Encinas et al.^58^ have recently reported that bacteria adhesion can be suppressed by submicrometer-scale surface roughness. Similarly, Jang et al.^59^ have incorporated nanotexture on stainless steel surfaces via electrochemical etching to inhibit bacterial adhesion.

To assess such paradoxical seeming trends of bacterial adhesion with respect to surface roughness observed in the literature, we have relied on hydrophobized quartz surfaces with uniformly controlled surface chemistry and coverage but systematically varying surface roughness. Fourteen different surface roughness values to cover roughness at multiple length scales were utilized for this investigation. The methyl terminated substrates were particularly selected due to their inert nature and since specific ligand–receptor interactions between such substrates and bacteria do not emerge. Hence, these surfaces allow us to clearly elucidate the influence of surface roughness on bacterial adhesion to abiotic surfaces that is mostly controlled by nonspecific interactions. As bacterial microorganisms, Gram-negative *Salmonella typhimurium* LT2 (*Salmonella*) and *Escherichia coli* O157:H7 (*E. coli*) as well as Gram-positive *Listeria innocua (Listeria)* have been utilized. With these selections, it is ensured that all bacteria have a similar shape (i.e., bacillus shape) and the effect of shape is not additionally superimposed to the influence of surface roughness. In addition, *Salmonella*, *E. coli*, and *Listeria* are three key microbial pathogens that are commonly associated with foodborne illnesses.^60,61^ While the characterization of surface chemistry and coverage was carried out using a scanning X-ray photoelectron spectroscopy microprobe (XPS), surface roughness was determined using atomic force microscopy (AFM). Bacterial adhesion was quantified using direct visualization via scanning electron microscopy (SEM).

## Experimental Methods

### Materials

Quartz (SiO_2_) was purchased from Ted Pella, Inc. (Redding, CA, USA). Tetrafluoromethane (CF_4_) and oxygen (O_2_) gas was obtained from Brazos Valley Welding Supply, Inc. and used directly as purchased (Bryan, College Station, TX, USA). Potassium hydroxide (98%, reagent grade) was purchased from Ward’s Science (Rochester, NY, USA). Trimethylsilyl chloride (TMCS, ≥ 95.0%) was obtained from Sigma-Aldrich Co. (St. Louis, MO, USA) and used with the hexane (≥95.0%) purchased from Avantor Performance Materials, Inc. (CenterValley, PA, USA). The 200-proof pure ethanol (HPLC grade) utilized was ordered from Koptec (King of Prussia, PA, USA). For bacterial experiments, tryptic soy agar (TSA), tryptic soy broth (TSB), and TSB containing 0.6% yeast extract (TSB-YE) were procured from Becton, Dickinson and Co. (Sparks, MD, USA).

### Preparation of Quartz Surfaces with Different Surface Roughness

Quartz slides were first rinsed with ultrapure water (resistivity ≥18.2 MΩ·cm) collected from a water purification system (Milli-Q Advantage A10; EMD Millipore Corp., Billerica, MA, USA) and then dried at room temperature. The dried slides were subjected to oxygen plasma treatment conducted in order to remove organic adsorbates using the CS-1701 reactive-ion etcher (RIE; Nordson MARCH, Concord, CA, USA). Following plasma treatment, the quartz slides were rinsed with Milli-Q water again and dried. Plasma treatment serves a dual-purpose: it not only increases the reactivity of surface groups but also inactivates any pre-existing bacteria on the surfaces.^62^ To produce surfaces with varying nanoroughness, the prepared quartz slides were treated in the CS-1701 reactive-ion etcher under CF_4_/O_2_ gas with varying etching times up to 2 h.

### Hydrophobization of Quartz Surfaces

To increase the hydrophobicity of the surfaces, the roughened quartz slides were modified using TMCS, which was prepared by diluting TMCS (6 wt %) in hexane. The quartz slides were then dipped into the TMCS and hexane solution for 24 h to allow the silanation modification of the surfaces to occur. Next, the samples were removed from the TMCS solution and rinsed three times with ethanol to remove excess TMCS and byproducts. At last, the samples were dried with compressed nitrogen gas before characterization.

### Characterization of Surface Roughness

The morphology and roughness of quartz surfaces with different roughnesses were studied using atomic force microscopy (AFM, Bruker Dimension Icon, Billerica, MA, USA) in tapping mode. Several parameters were quantified to analyze the surface roughness, such as the root-mean-square (RMS) roughness, autocorrelation length, and roughness ratio. The RMS roughness was calculated based on the root-mean-square of the height of microscale peaks and valleys as a means of quantifying the average feature size. The roughness ratio was calculated by dividing the actual surface area to the projected area (φ ≥ 1). Autocorrelation length was obtained from the analysis of power spectral density function (PSDT). The Gwyddion software 2.49 (Czech Metrology Institute, Jihlava, Czech Republic) was used to analyze the AFM images and calculate the above-mentioned parameter values for each sample.

### Characterization of Surface Chemistry

The chemical interactions between the modified quartz and TMCS were studied via attenuated total reflectance-Fourier transform infrared spectroscopy (ATR-FTIR) using the IRPrestige-21 (Shimadzu Corp., Kyoto, Japan) system. The results of ATR-FTIR were analyzed with the IRsolution version 1.40 (Shimadzu Corp., Kyoto, Japan) software. TMCS coverage on the modified surfaces was characterized utilizing a PHI VersaProbe II scanning XPS microprobe (Physical Electronics, Chanhassen, MN, USA) The ATR-FTIR and XPS results are shown in the Supporting Information to indicate the successful modification of the surfaces as well as the similar chemical properties and coverage of the surfaces (Figure S1 and Figure S2)

### Preparation of Bacterial Cultures

Working cultures of Gram-negative bacterium *Salmonella enterica* subsp. *enterica* serovar Typhimurium str. LT2 (*Salmonella*, ATCC 700720) and *Escherichia coli* O157:H7 (*E. coli*) as well as Gram-positive *Listeria innocua* NADC 2841 were prepared following our previously described method.^63−65^ Briefly, bacteria were separately transferred by microloops (10 μL) from TSA slants to 9.0 mL TSB solutions. After aerobically incubating at 37 °C for 24 h, a second transfer of each bacterial strain was carried out by transferring one 10 μL loop of material from each of the first solutions to 9.0 mL of fresh TSB followed by incubation under the same conditions. Afterward, the working culture of each bacterium was purified by centrifuging at 1500 × *g* for 15 min and resuspending in 0.1 wt % aqueous peptone solution. This was performed three times for each working culture. The final concentration of each working culture was determined through plate counting to be 8.6 ± 0.3 log_10_ CFU/mL, 8.8 ± 0.2 log_10_ CFU/mL, and 9.1 ± 0.2 log_10_ CFU/mL for *E. coli*, *Salmonella*, and *Listeria*, respectively. Right before performing bacterial inoculation assays, the bacterial cells were suspended in sterilized deionized (DI) water.

While the media suspending bacteria is often chosen to be PBS buffer in medical microbiology, many engineering applications and situations in different fields do not involve such a buffer (e.g., environmental surfaces in hospitals, engineering surfaces in fresh water, washing of vegetables and fruits with water, irrigation of crops, freshwater flow in pipelines). In this study, we focus on the cases where bacteria are not suspended in a buffer. Electrical conductivity measurements on these suspensions revealed that the electrical conductivity of *E. coli*, *Salmonella*, and *Listeria* suspensions was 5.6 ± 2.7, 7.9 ± 0.3, and 6.1 ± 0.3 μS/cm, respectively. These conductivity values correspond to the equivalent salt (NaCl) concentration of 39.1 ± 5.5, 57.4 ± 0.7, and 43.2 ± 0.6 μM, respectively, which are likely byproducts of lysed cells and bacterial metabolites. The long-term preservation and survivability of the *E. coli, Salmonella*, and *Listeria* were confirmed, and it was found that a less than 0.5 log unit reduction of the bacteria population occurs after storing such bacteria in water over 1 week.^66^ The frequent bacterial contamination scenarios with freshwater in many engineering applications and survival of bacteria under these conditions (non-PBS conditions) are the rationale behind the selection of bacterial inoculation conditions in this study.

### Characterization of Bacteria

The zeta potential of bacteria was evaluated using electrophoretic light scattering (Zetasizer Nano, Malvern Panalytical, Malvern, United Kingdom). In these measurements, the bacterial concentrations of 0.1–1 vol % suspended in deionized (DI) water were used. The interfacial tension of bacteria was determined by preparing dehydrated bacterial lawn on silicon wafers. First, the bacterial suspension prepared as described above was washed with DI water and centrifuged. Afterward, the supernatant was poured off and the lower layer of bacteria cells were transferred to the <100> silicon wafers by using a sterile spatula and carefully spread out by an L-shape spreader to generate a uniform distribution. The samples were left to dry at room temperature for 12 h to gain a dehydrated, homogeneous bacterial surface with a stable contact angle.^67^ SEM images was taken to confirm the surface bacterial coverage (Figure S3). The static contact angles of bacteria were evaluated using milli-Q water and diiodomethane (DIM) via a sessile drop technique where the droplet volume was about 5 μL. The contact angle results were analyzed by ImageJ software (National Institutes of Health, Bethesda, MD, USA) via Low-Bond Axisymmetric Drop Shape Analysis (LBADSA).^64^ The surface tension of each bacterium was calculated based on the harmonic mean and geometric mean equations, using the contact angles of water and DIM.

The cell size of each bacterial strain used in this work was studied by AFM (Dimension Icon AFM, Bruker, Billerica, Massachusetts) and SEM (JEOL JSM-7500F, JEOL Ltd., Tokyo Japan). To ensure the reliability of the data, three AFM and SEM images with multiple bacteria each were analyzed to determine the cell length and width. AFM micrographs were analyzed with by Gwyddion software 2.49 while SEM images were analyzed with ImageJ software 1.8.0_172 (ImageJ, U.S. National Institutes of Health, Bethesda, Maryland, USA). Representative AFM images for single bacterium are shown in Figure S4 and the interfacial and morphological characteristics of bacteria are summarized in Table 1.

**Table 1:** Summary of Structure and Interfacial Characterization of the Bacteria Used

	*Salmonella*	*E. coli*	*Listeria*
Type	Gram-negative	Gram-negative	Gram-positive
Dimensions (μm)	Length: 2.14 ± 0.27	Length: 1.94 ± 0.15	Length: 1.37 ± 0.20
	Width: 0.92 ± 0.11	Width: 1.00 ± 0.11	Width: 0.59 ± 0.07
Contact angle of water (deg)	21.9 ± 3.0	29.8 ± 2.6	19.4 ± 0.2
Contact angle of DIM (deg)	47.6 ± 1.4	38.1 ± 0.3	42.2 ± 1.2
Surface tension (mN/m)	70.9 ± 1.4	70.0 ± 1.1	73.0 ± 0.30
Zeta potential (mV)	–21.6 ± 5.4	–9.8 ± 3.7	–25.1 ± 9.6

### Inoculation Assay

Bacterial suspensions, prepared as described above, were used directly to inoculate the methylated quartz samples of varying roughnesses. The samples were each submerged in a 9.0 mL bacterial suspension and shaken by a mini shaker (VWR International, LLC, Radnor, PA, USA) at 150 rpm for 4 h. After 4 h of shaking, each sample was removed from the suspension and held vertically for 5 min to remove any remaining liquid droplets to reduce drying effects on the surfaces. Afterward, the surfaces with attached bacteria were gently blown with sterile nitrogen gas to further remove any liquid. As a whole, this assay involves an initial bacterial deposition step of 4 h followed by a weak detachment step associated with the passage of air–liquid interface over the weakly (reversibly) attached bacteria. A summary table of the experimental matrix can be found in the Supporting Information (Table. S1).

### Enumeration of Bacterial Adhesion

The direct counting method was performed to quantify the number of attached bacterial cells on each sample using SEM imaging. Before SEM imaging, the bacterial cells were inactivated by exposing them to small amounts of acrolein vapor. Following inactivation, the sample surfaces were coated with a thin film of palladium and platinum (Pd/Pt) alloy to increase surface electrical conductivity during SEM imaging. Here, the presence of thin metal coating serves a side function of immobilizing and fixating adherent bacteria on the substrate. To increase statistical reliability of the quantification of the attached bacteria, at least ten different areas of each sample were randomly selected to be imaged and quantified directly through SEM. The SEM studies were performed at a pressure of 7.2 × 10^–7^ Torr, working distance of 15 mm, and operation voltage of 5 kV. The bacterial numbers were counted manually (to distinguish overlapping and aggregated cell cases) and ImageJ via Cell Counter Plugin.

### Statistical Analysis

In order to determine the statistically differing counts of bacterial attachment between the samples with different roughness, the bacterial density for each microorganism was statistically analyzed via one-way analysis of variance (ANOVA) with Tukey’s posthoc test. The *p*-value for statistical difference between means was set as *p* = 0.05. All statistical analyses were conducted by using JMP software (SAS Institute, Inc., Cary, NC, USA).

### Calculation of DLVO Interactions between Bacterium and Rough Surface Using Surface Element Integration (SEI) Method

**Figure 1:**
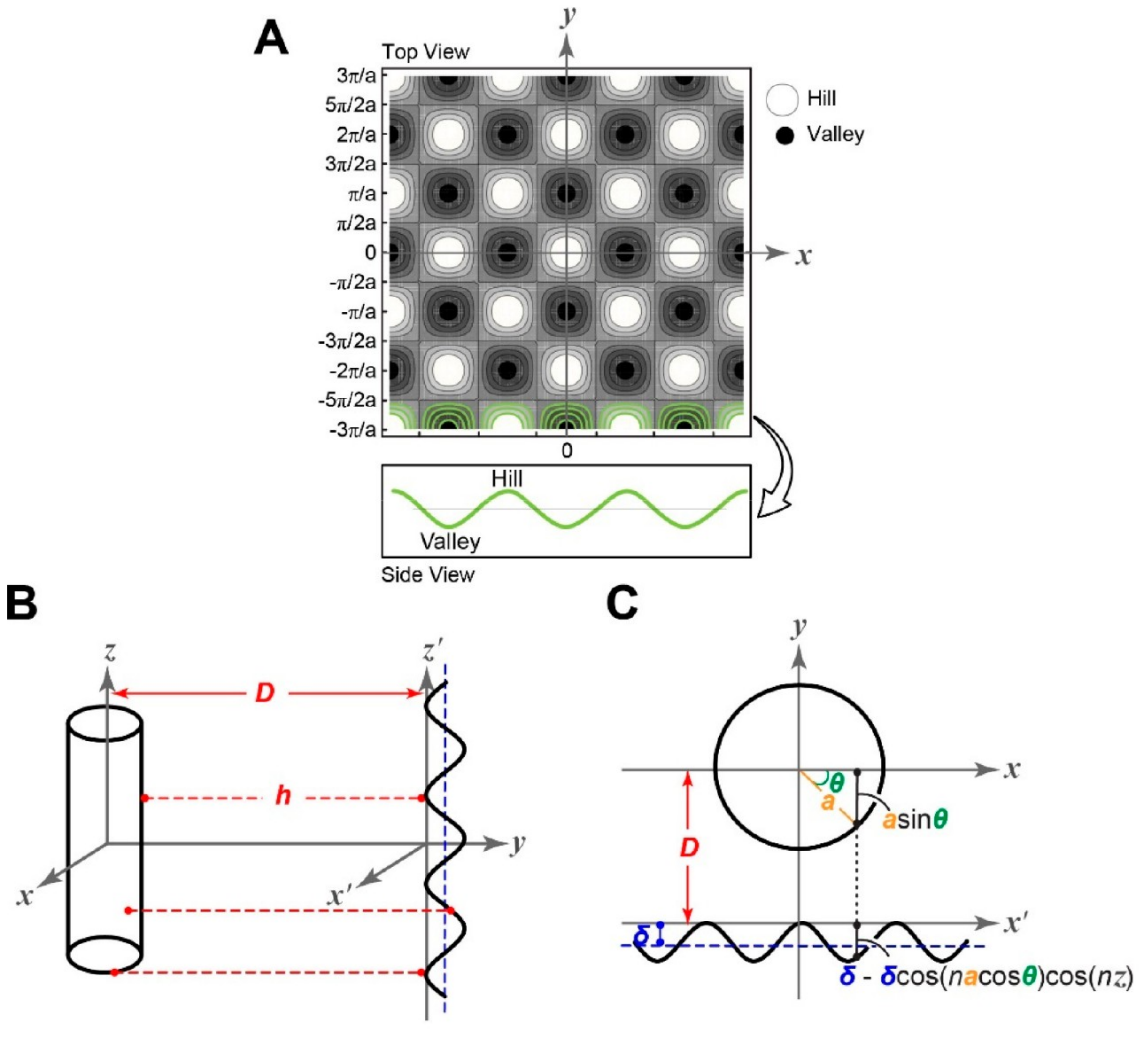
Schematic illustrations of the model for the simulation of bacteria and rough surfaces interactions. (A) Schematic illustration of the model surface geometry used in calculating interaction between a bacterial cell and a rough surface via the SEI method. (B) The cylinder-fixed coordinates and the surface-fixed coordinates have different origins but the same axis directions. The distance, *h*, means the closest distance between the cell surface and quartz surface, calculated by adding the distance from cell surface to the x′-axis and the distance from x′-axis to the rough surface. (C) The separation, *D*, indicates the distance between the center of cylinder and the peak of the double-cosine function.

The interactions between a rough bacterium and a surface were calculated using the surface element integration (SEI) method. The method was developed by Bhattacharjee and Elimelech^68^ for calculating the DLVO interactions for complex geometries where obtaining a solution for the nonlinear Poisson–Boltzmann equation is complicated and computationally expensive. In this work, we have implemented the SEI method to gain insights into the bacterial interactions on rough surfaces. The roughness was modeled by the double-cosine function [*cos*(*x*) cos(*y*), which is a periodic, 2-D wave] as shown in Figure 1. In this analysis, since the size of the bacteria is much larger than the wavelength of double-cosine function (i.e., characteristics roughness scale), the interaction calculation is insensitive to the exact positioning of bacteria relative to bacteria (i.e., the center of bacteria directly projected above a peak, maximum, versus projected above a valley, minimum).

## Results and discussion

### Characterization of Surface Roughness

**Figure 2:**
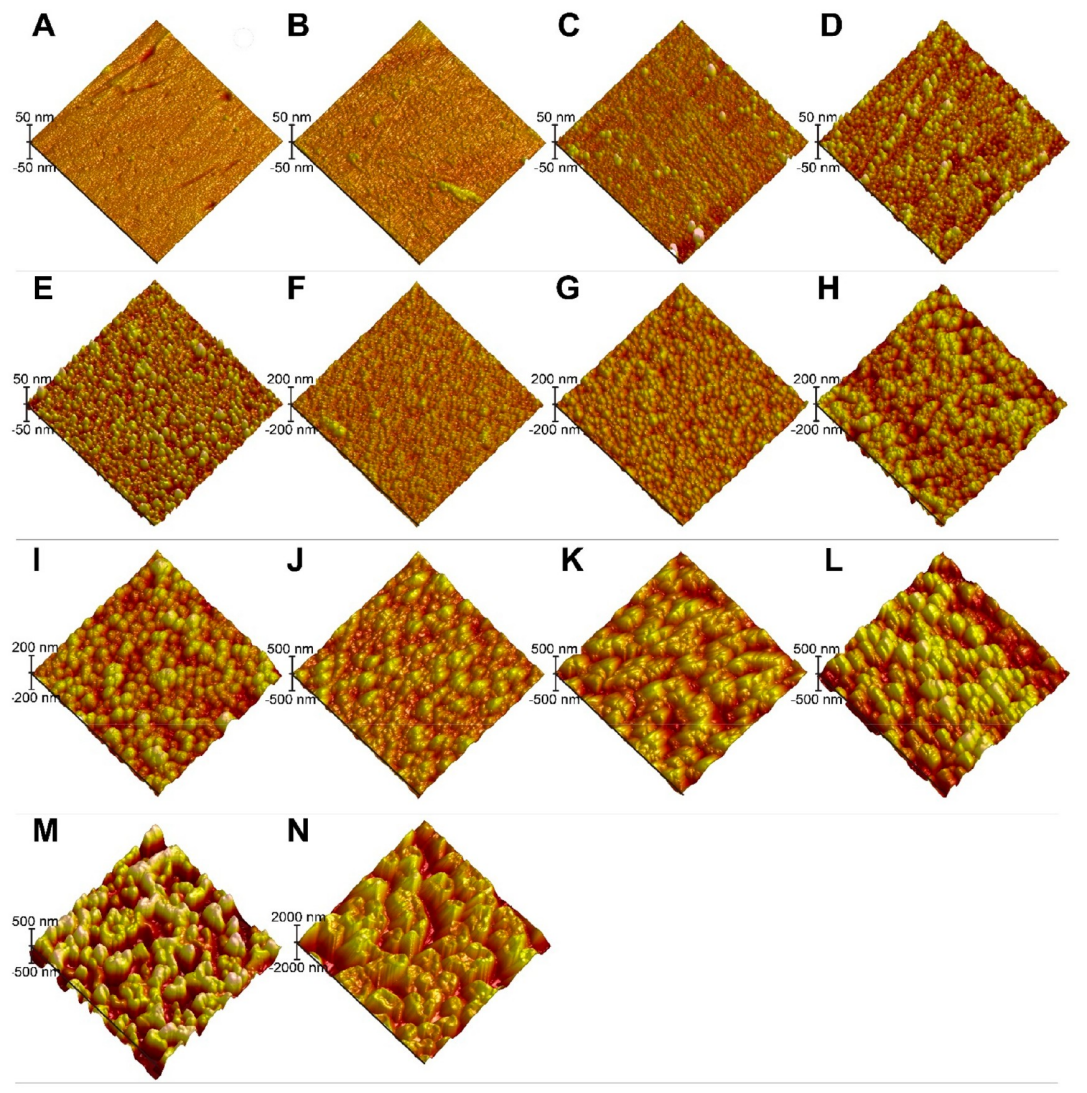
AFM micrographs of methylated quartz surfaces of systematically varying roughnesses (A-N). The samples were labeled alphabetically in accordance with increasing etching times (and increasing root-mean-square, RMS, roughness) used in their preparation as described in Table 2. For these samples, the RMS roughness progressively ranges from 1.7 to 385 nm. For each condition, three to seven samples were prepared using identical etching processes. All AFM micrographs have the same scan area of 5 μm × 5 μm. On the left side of each micrograph, the corresponding scale bar for the height is shown.

Figure 2 shows AFM micrographs of 14 different methylated quartz samples prepared by varying the etching time with CF_4_/O_2_ gas (Samples A-N). At short plasma etching times (<5 min), the features of the polished quartz samples, having an RMS roughness of 1.7 nm, gradually eroded. Isolated, nanoscale hemispherical features started to emerge on the surfaces (Figure 2C-E). As the etching times were increased (10 to 25 min), the areal density of nanohemispheres increased (Figure 2 F-G). At larger etching times (30 to 120 min), the nanohemispheres transformed into ovoid textures (Figure 2 I-L) and then cratered, volcano-like textures (Figure 2 M-N). Through these morphological transitions, an RMS roughness range of ∼2 nm to ∼390 nm was covered.

Surface roughness can be described in terms of various parameters. In the context of analyzing/correlating bacterial adhesion trends with roughness, amplitude parameters (e.g., root-mean-square, RMS roughness), lateral parameters (autocorrelation length), and areal roughness parameters (e.g., roughness ratio) were selected by considering the interplay among characteristic lengths of bacteria (diameter and length) and characteristic lengths of surface roughness. First, RMS roughness indicates the effective height of roughness asperities of a surface. The comparison of bacterial diameter and RMS roughness indicates what fraction of adhering bacteria could be embedded into surface texture. Second, the spacing between asperities is the key length scale controlling whether a bacterium can dock-in between surface asperities. If the interasperity spacing is too small, bacteria must reside on top of the asperities, which implies reduced van der Waals interactions and an unfavorable bending energy for bacteria to accommodate into these spaces. Autocorrelation length is a measure of periodicity and directly related to the interasperity spacing. Third, the roughness ratio indicates the increase in the effective area of a surface compared to the projection area. This means a high roughness ratio can indicate a higher number of sites for bacteria to adhere assuming bacteria can conform to the roughness. Table 2 lists the key surface roughness characteristics and static contact angles of water on these methylated quartz surfaces. The prepared samples covered RMS roughness values from ∼2 nm to ∼390 nm while the roughness ratio ranged from 1.0 to 3.7 for these surfaces. The smallest and largest value of autocorrelation length was ∼27 nm to ∼118 nm, respectively. RMS roughness and roughness ratio exhibited a correlation with etching time (i.e., increased etching time results in increased RMS roughness and roughness ratio), whereas the autocorrelation function has up and down trends with respect to the etching time.

**Table 2:** Surface Characteristics and Wetting Properties of the Prepared Methylated Quartz Surfaces

sample	etching time (s)	RMS roughness (nm)	roughness ratio	autocorrelation length (nm)	contact angle of water (deg)
A	0	1.7 ± 0.1	1.00 ± 0.00	51.5 ± 9.3	95.5 ± 1.2
B	15	3.0 ± 1.6	1.00 ± 0.00	56.9 ± 11.7	97.3 ± 1.6
C	30	5.8 ± 1.2	1.01 ± 0.01	35.5 ± 7.1	98.2 ± 1.2
D	60	9.1 ± 1.7	1.03 ± 0.02	47.5 ± 10.6	106.5 ± 1.2
E	300	11.3 ± 2.3	1.07 ± 0.03	27.1 ± 11.3	117.7 ± 1.7
F	600	16.8 ± 1.1	1.13 ± 0.08	34.0 ± 3.6	128.6 ± 1.4
G	900	26.9 ± 3.5	1.16 ± 0.03	52.3 ± 3.8	143.3 ± 1.9
H	1200	44.9 ± 5.1	1.19 ± 0.01	64.6 ± 5.5	151.1 ± 1.4
I	1500	54.8 ± 9.2	1.36 ± 0.03	75.7 ± 10.2	151.1 ± 0.7
J	1800	63.7 ± 5.7	1.37 ± 0.04	115.7 ± 6.6	152.8 ± 1.3
K	3600	85.7 ± 8.7	1.37 ± 0.02	106.6 ± 9.2	151.4 ± 1.1
L	4800	128.7 ± 18.0	1.81 ± 0.18	81.0 ± 4.2	149.1 ± 1.1
M	6000	215.0 ± 16.1	2.83 ± 0.57	118.7 ± 8.7	149.4 ± 1.3
N	7200	385.3 ± 110.4	3.69 ± 0.51	128.0 ± 10.4	150.8 ± 0.9

The contact angle of water demonstrated a large variation with respect to the surface morphology. While the static contact angle was 95–97° for smooth surfaces, the wetting behavior transitioned into a superhydrophobic behavior (i.e., contact angle of water >150°) for surfaces with RMS roughness greater than ∼55 nm.

### Influence of Surface Roughness on Adhesion of *Salmonella*

**Figure 3:**
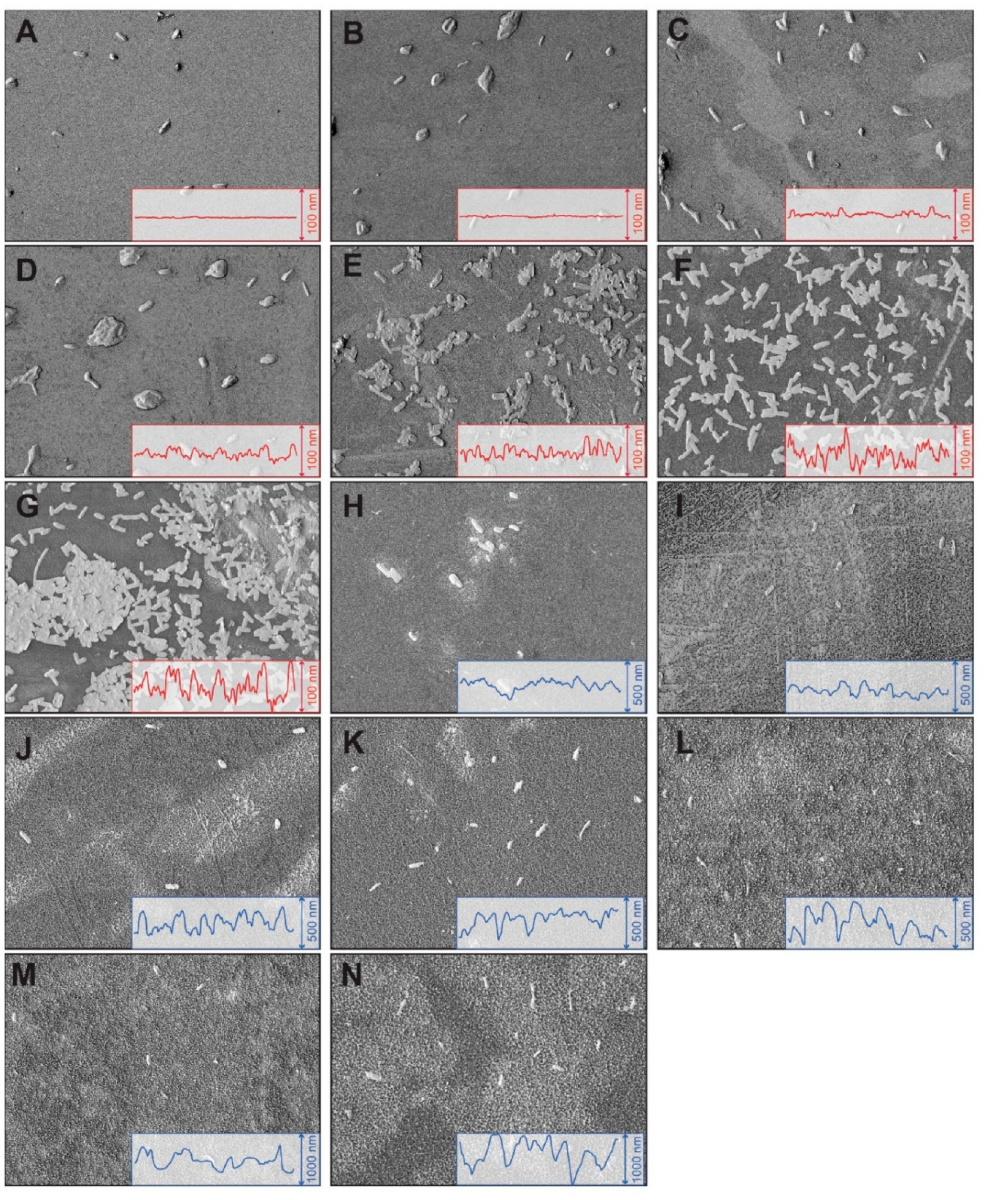
SEM micrographs displaying bacterial adhesion trends on hydrophobically modified quartz surfaces roughness for *Salmonella*. (A-N) The surface roughness RMS varies systematically. The frame at the right lower corner in each figure shows the cross-section profile of the surface, reflecting different surface roughness. The height of frame is denoted as scale bar (100, 500, and 1000 nm, respectively). Bacterial adhesion is significantly lower for superhydrophobic surfaces (samples H-L) compared to other samples (*p* < 0.05). Each micrograph has the same size of 45.6 μm × 60.7 μm (see Figure S5 in Supporting Information for larger images).

After extensively characterizing and confirming the uniformity of the surface chemistry and coverage on the prepared surfaces of varying roughness, the samples were fully immersed in bacterial (*Salmonella*) suspension for 4 h to gain insights into bacterial adhesion trends at the early stage of biofilm formation. It was found that the surface roughness strongly influenced the extent of bacterial adhesion on the surfaces (Figure 3). At low roughness values (i.e., RMS < ∼10 nm), the surfaces contained isolated microcolonies with a relatively low number of adherent bacteria and a low overall areal density (Figure 3 A-D). In addition, the presence of extracellular polymeric substances (EPS) around microcolonies was also noted. At intermediate roughness values (i.e., RMS between 10 and 40 nm), the number of adherent bacteria on the samples increased significantly and the isolated microcolonies were replaced by loosely connected bacterial monolayers (Figure 3 E-G). In addition, bacteria appeared to be more deformed/flattened on these surfaces, indicating a stronger attraction between bacteria and such surfaces. At high roughness values (i.e., RMS roughness >45 nm), the areal density of adhering bacteria was extremely low and no microcolonies were observed. Bacteria mostly resided as single, isolated organisms on these surfaces while a small fraction was in the form of dimeric and trimeric aggregates (Figure 3 H-N).

### Adhesion Response of Different Bacterial Species to Surface Roughness

**Figure 4:**
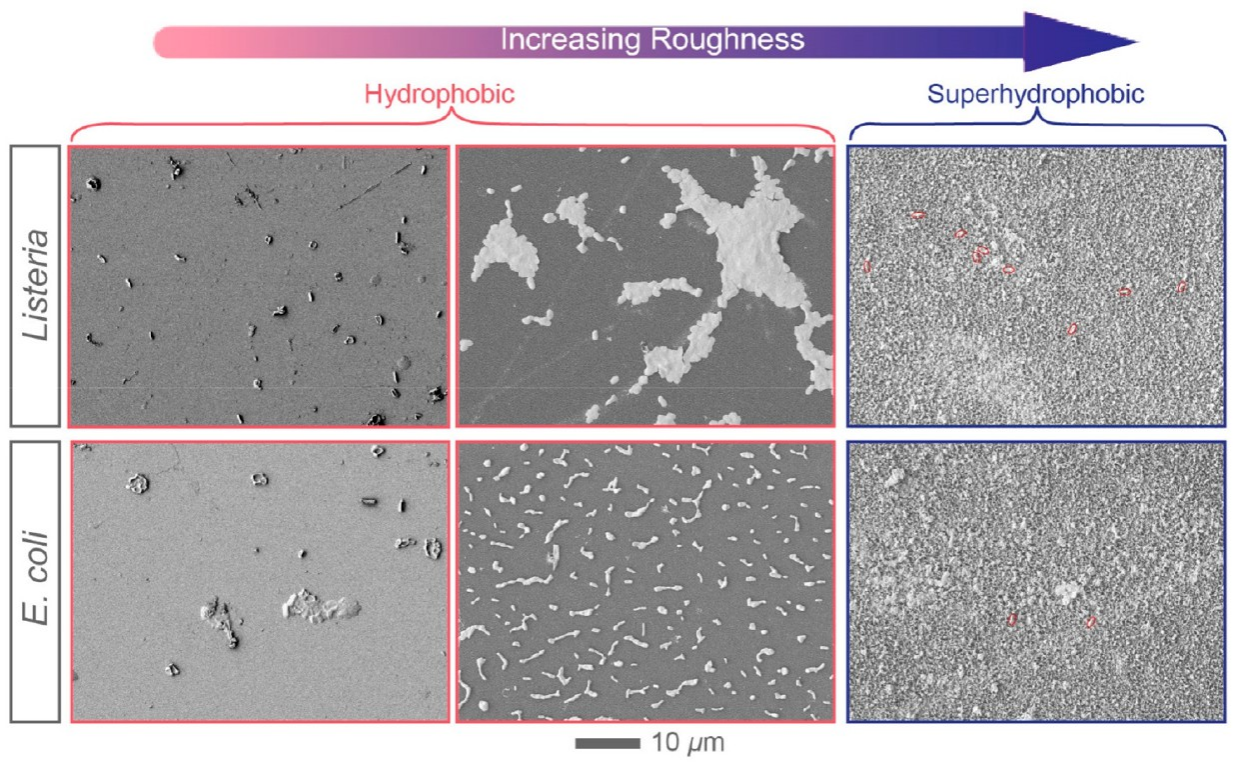
SEM micrographs displaying bacterial adhesion trends on hydrophobically modified quartz surface roughnesses for *Listeria and E. coli*. The surface roughness RMS varies systematically. Bacterial adhesion is significantly lower for superhydrophobic surfaces, where the bacterial cells are marked with red ovals, compared to other samples (*p* < 0.05). The scale bar is the same for all micrographs and is equal to 10 μm. (See Figure S6 in Supporting Information for larger images.)

Aside from *Salmonella*, we have also utilized *Listeria* (Gram-positive) and *E. coli* (Gram-negative) to determine if the dependence between surface roughness and bacterial adhesion is similar for different types of bacteria. Figure 4 demonstrates *Listeria* and *E. coli* adhesion trends on methylated quartz substrates of varying surface roughness. Three typical samples (corresponded to D, G, N in Figure 2 and Table. 2) were chosen to study the different adhesion behavior on three types of surfaces, relatively smooth surface (hydrophobic), moderately rough surface (hydrophobic), and rough surface (superhydrophobic). Similar to the case of *Salmonella*, at low surface roughness, the bacterial densities, Γ, were low, 2–3 × 10^–2^ cells/μm^2^. At intermediate roughness values, the number of adhering bacteria increased to 5–10 × 10^–2^ cells/μm^2^. On the other hand, when surface roughness reached high values (when the surfaces became superhydrophobic), the bacterial adhesion was significantly hindered and had a range of 2–9 × 10^–3^ cells/μm^2^. Based on these results, the statistical analysis with Tukey-Kramer honest significance test revealed that the bacterial adhesion is not significantly different for these three types of bacteria at a given surface roughness (*p* > 0.05, see Supporting Information Table S2 for further analysis). While the number density of adhering bacteria is similar, the colonization behavior showed some differences with respect to microorganism type. For instance, at intermediate roughnesses, *Listeria* tended to form tightly packed colonies whereas *E. coli* aggregated into dimers, trimers, and tetramers with a characteristic separation distance of 3–5 μm (Figure 4). In addition, the nonwetting behavior of the EPS layer and relatively large volume of EPS bridges between *E. coli* was evident.

There can be multiple reasons responsible for the differences in microcolony formation behavior. While *Listeria* is a Gram-positive bacterium, *E. coli* is a Gram-negative bacterium. For Gram-negative bacteria, there exists three principal layers in the cell wall: the outer membrane, the peptidoglycan cell wall, and the cytoplasmic or inner membrane. The Gram-positive wall differs from the Gram-negative wall in several ways: (i) The outer membrane is absent in Gram-positive bacteria. (ii) Gram-positive bacteria are covered with a peptidoglycan layer much thicker than the peptidoglycan layer of Gram-negative bacteria. (iii) Gram-positive bacteria tend to contain teichoic acids, which can be wall teichoic acids coupled to peptidoglycan and lipoteichoic acids anchored to the cell membrane. Their acidic nature results in a negative surface charge in aqueous media, which can modulate the electrostatic double-layer forces differently. In addition, the difference in quorum sensing behavior of Gram-positive and Gram-negative bacteria, Gram-negative bacteria rely on n-acyl homoserine lacton molecules (autoinducer-1, AI-1) while Gram-positive bacteria use mainly peptides (autoinducer peptides), can lead to differences in chemotaxis, which is related to the microcolony formation as well. Furthermore, the surface topography also has an effect on the bacterial adhesion behavior in terms of morphological, genomic, and proteomic response, while the response of different types of bacteria may vary.^69^ It was observed that the type-1 fimbriae disappeared in *E. coli* adherent onto nanostructured substrates, which were believed to affect its colonization behavior, the regulation of proteins involved in the adhesion process, and defense mechanisms.^70^ The *Pseudomonas aeruginosa* cells, on the other hand, were found to lose their flagella upon adhering to a nanostructured surface.^69^ From a purely hydrodynamics perspective, the aspect ratio of *E. coli* was ∼1.9 while that of *Listeria* was ∼2.3. Since nonspherical colloids can rotate as it translates; the anisotropic translational diffusive motion of rod-shared bacteria can differ significantly from that of a sphere (cocci) and a larger aspect ratio also translates into a further deviation from a spherical shape. The hydrodynamic theory by Doi and Edwards^71,72^ predicts that for rods (irrespective of the aspect ratio) under the no-slip (stick) boundary condition, the translational diffusion coefficient in the parallel (*D*∥) direction (parallel to its major axis) is twice of that in the perpendicular direction (*D*⊥). On the other hand, under a slip boundary condition, there exists a decoupling between the parallel and perpendicular motion, and the ratio of the diffusion coefficients in the parallel and perpendicular direction approaches the aspect ratio.^73,74^ The surface characteristics of different microorganisms (peptidoglycan exterior versus lipidic exterior) may lead to a different slip or partial slip behavior in aqueous media in accordance with the degree of favorable interactions with water molecules. Such secondary effects can also alter the bacterial adhesion behavior when the overall process is diffusion-controlled as opposed to “reaction-controlled”. The different colonization behavior observed can be attributed to the combination of the above-mentioned factors.

### Bacterial Reaction/Deposition Kinetics Analysis: Hydrophobic Regime

To explain the observed bacterial adhesion trends, we consider a reaction/deposition kinetics scheme where the adhesion of bacteria onto the substrate is modeled as an irreversible, first-order reaction/deposition (process):^75,76^
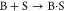
1where *B* denotes planktonic bacteria, S denotes effective surface area permitted for bacterial adhesion, and B·S indicates adherent bacteria. The corresponding expression for the reaction (physisorption) rate, *r*, and the change in the concentration of entities relevant to the system is
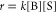
2
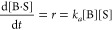
3where [B·S] is the surface concentration of adherent bacteria, Γ (#/m^2^), [B] is the bacterial concentration in suspension (#/m^3^), [S] is the effective available surface area (m^2^) and *k*_*a*_ is the reaction constant that varies with the surface topography (1/m·s). Since only a small fraction of bacterial suspension accumulates as adsorbates on the surface, the bacterial concentration in the suspension can be assumed constant. Because the effective available surface area decreases as bacterial adhesion takes place, it can be calculated from [S] = [S]_T_(1 – θ × [B·S]) where [S]_T_ is the total initial surface area and θ is a projected contact area per bacterium. Then, the integration of eq 3 results in the following expression for [B·S]:
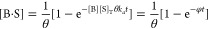
4where φ = [B][S]_T_*θk*_*a*_.

Given that all bacterial inoculation experiments were carried out at an inoculation time of 4 h and the same bacterial concentration, [B], differing number of bacteria on different surface roughness can be explained either by the modification of effective surface area [S]_T_ or the change of bacterial adhesion rate constant, *k*_a_, or the combination of both factors. We have divided our discussion related to these concepts into two parts: hydrophobic regime and superhydrophobic regime. For the hydrophobic regime, our analysis first focuses on the effect of increased surface area due to roughness on bacterial adhesion. Given that bacteria surface is deformable, the existence of surface roughness implies a conformal contact between bacteria and the surface. However, since the elastic energy needed to stretch the bacteria wall from its equilibrium position is unfavorable compared to just bending and buckling the bacterial surface, the change in the total surface area of bacteria wall is expected to be small.^77−79^ Hence, the increase in the substrate area can increase the nominal number of bacteria per unit projected area.

**Figure 5:**
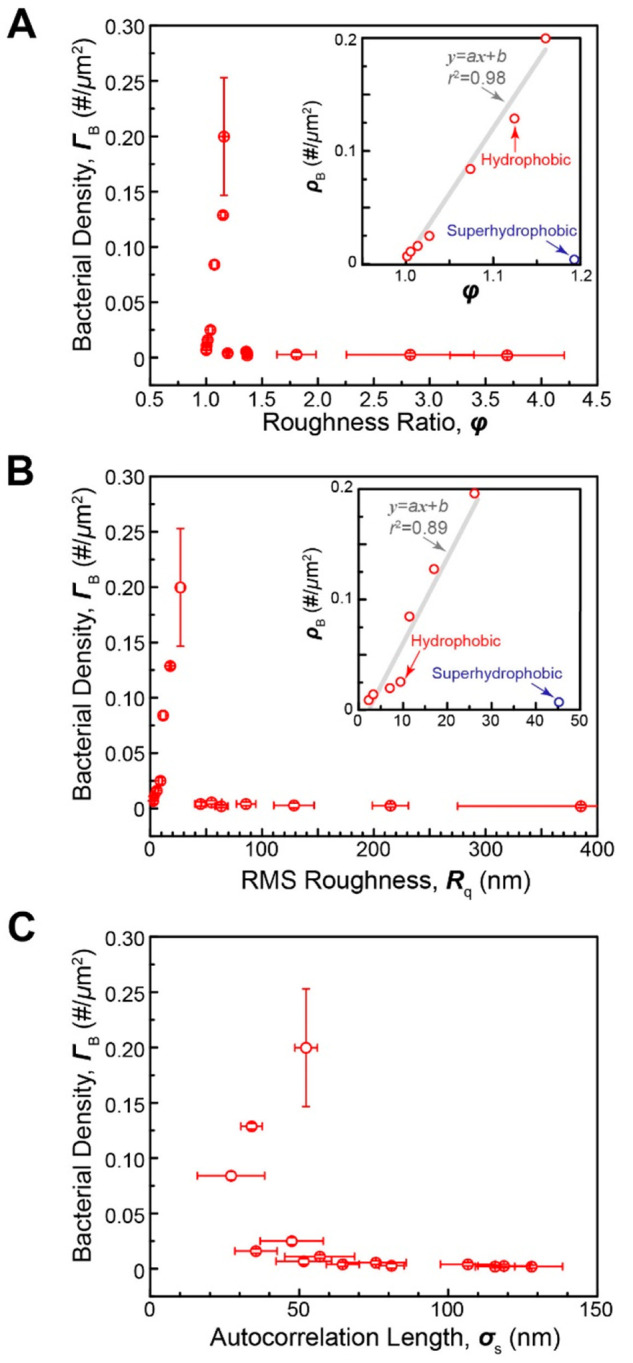
Correlations between bacterial adhesion density (*Salmonella*) and various variables. (A) Roughness ratio. (B) RMS roughness. (C) Autocorrelation length. Insets focus on the correlations for the hydrophobic regime (by omitting superhydrophobic regime).

To understand the influence of increased effective surface area, we plotted bacterial adhesion with respect to roughness ratio (Figure 5). We found that there was a linear relationship between roughness ratio and bacterial adhesion, with the coefficient of determination, *r*^2^, of 0.98. On the other hand, our attempts to correlate bacterial adhesion with RMS (*Rq*) roughness led to a poorer fit with *r*^2^ < 0.90. For the case of autocorrelation length, there was no clear trend between bacterial adhesion and autocorrelation length. Here, we must note that for all roughnesses studied in this work, bacterial size was greater than the height and spacing length scales of the surface features. The trends may change once bacteria are smaller than interasperity distance. Overall, considering that the roughness ratio is the ratio of actual surface area and projected surface area, such a linear correlation suggests that bacterial adhesion is directly controlled by surface area.

Regarding the rate of bacterial adhesion, for a colloidal system (e.g., bacteria), Kramers’ rate theory^80,81^ can be used to estimate the adsorption/adhesion rate constant in terms of diffusivity, *D*:^82^
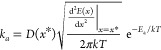
5where *D*(x*) is the diffusivity at the distance leading to the maximum in the potential energy profile, *k* is the Boltzmann constant, *T* is temperature, and *E*_a_ is the activation barrier for adhesion. For bacterial systems, the activation energy, *E*_a_, is governed by the interplay among electrostatic double-layer forces and van der Waals interactions as described by the DLVO interactions or extended DLVO.^83−87^ For the cases where the diffusivity is mostly constant (i.e., the wall and interfacial effects on diffusivity is negligible), Kramers’ rate theory can also be expressed in a slightly different form:^88^
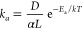
6where α is the width of the potential at a distance *kT* below the maximum, and *L* is the distance that a bacterium in the bulk state needs to travel to reach the barrier maximum. As a first approximation, *L* can be equated to the interbacterial spacing set by the planktonic concentration of bacteria in the suspension. A simpler argument can also be made with an Arrhenius-type expression as well, while the validity of Kramers’ model is known to be better for colloidal systems.

**Figure 6:**
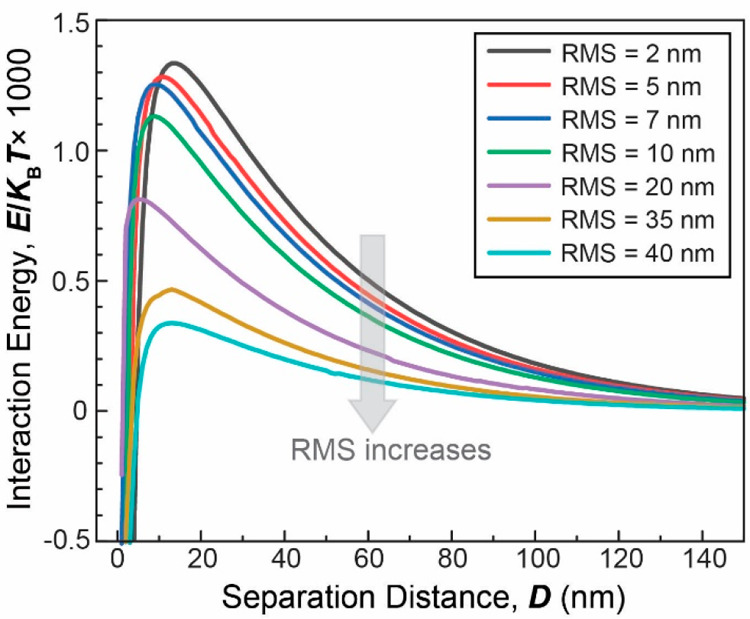
DLVO interaction energy profile between a *salmonella* cell and methylated quartz surfaces with different roughness. The parameters used to obtain the plot was as follows: Hamaker constant, *A*_bws_ = 3.29 × 10^–20^ J; bacterial length = 2.14 μm, bacterial diameter = 0.92 μm; zeta potential of bacteria (*Salmonella*) = −21.6 mV; zeta potential of surface (methylated quartz) = −20.0 mV; Debye length = 40.5 nm; and temperature = 298.15 K.

Previous streaming potential and surface science studies reported that a methylated quartz surface bears a negative zeta potential due to the specific organization and orientation of hydroxyl ions onto hydrophobic interfaces and the dissociation of uncovered silanol and siloxane groups of quartz in aqueous media.^76,89^ For such surfaces, the energetics of bacterial adhesion is mainly governed by the DLVO interactions where the interplay among van der Waals attraction (VDW) and electrical double layer repulsion (DL) controls the range and magnitude of interactions. The balance between these interactions also controls the height of the activation barrier for the process of bacterial approach toward a surface.^90^ Using the surface energy integration (SEI) method (see Supporting Information for detailed explanation), we have obtained the energy-distance profiles for the bacterium-surface system at varying roughness values comparable with the experimentally observed roughness values (Figure 6). It was found that increasing surface roughness decreases the repulsion and the activation barrier for adhesion, *E*_a_, in a linear fashion (*r*^2^ = 0.995, Figure S7). In other words, as the surface roughness increases, the activation energy for bacterial energy decreases, indicating more favorable conditions for bacteria to attach to the surfaces. Clearly, this analysis is somehow simplified given that bacteria can freely rotate in aqueous media. Rod-shaped bacterium can change its orientation as it approaches a surface. Furthermore, *Salmonella* has surface appendages which can facilitate their docking to surface and overcome the activation energy barrier. Hence, only general trends of how the activation energy depend on surface roughness rather than the magnitude of interaction energy should be considered in this analysis.

Overall, based on the above-mentioned analysis, both increased surface area with increasing roughness and decreased activation energy with increased surface roughness can enhance the extent of bacterial adhesion. It is difficult to deconvolute their relative importance with the existing experimental data. However, it can be deduced that since the rate constant has an exponential dependence to the activation energy and the activation energy is linearly decreasing with roughness, the rate constant, *k*_a_, is expected to more strongly control the extent of bacterial adhesion compared to increased surface area of adhesion, [S]_T_ in light of eq 4. This concept can be supported by the data published by Yoshimoto et al.^91^ They showed that for glass surfaces having the same optical grade roughness, increasing ionic strength from 5 mM to 20 mM and from 20 mM to 50 mM, caused the relative bacterial (*Acinetobacter* sp. Tol 5) adhesion on glass to change from ∼0.5 to ∼0.7 and to ∼1.2.

### Bacterial Reaction/Deposition Kinetics Analysis: Superhydrophobic Regime

For surfaces with contact angles above 150° (i.e., superhydrophobic surfaces), the areal density of adhering bacteria was much less. Here, the presence of interstitial air (air gaps) as described by Cassie–Baxter model must be considered while assessing the bacterial adhesion trends on surfaces of varying roughness. Namely, in this regime, air gaps can cause some changes in the way a bacterium can adhere on a rough surface, thus both surface roughness and wetting influence the bacterial adhesion. First, aqueous media suspending planktonic bacteria will only be in contact with the upper sections of asperities for superhydrophobic surfaces. Second, the replacement of water with air in the valley between asperities will modify the van der Waals interactions between a bacterium and the surface. Third, the presence of air gaps also implies the existence of Laplace pressure force acting toward bacteria. We now consider these effects in detail.

Since the horizontal and vertical roughness length scale is still smaller than bacterial size, the attachment of a bacterium on a superhydrophobic surface implies that the bacterium resides on top of the surface asperities similar to the hydrophobic case. However, a careful inspection of SEM micrographs in Figure 3 (A-F versus H-L) indicates that the effective projected area of bacteria on superhydrophobic surfaces is smaller than that on hydrophobic surfaces, and the thickness of bacteria was larger on superhydrophobic surfaces, indicating poorer spreading/wetting of bacteria on the surface. This trend could be ascribed to the larger roughness values of superhydrophobic surfaces and the resultant weakening of attractive van Waals forces due to the larger effective distance between a bacterium and a surface. It is important to underline that while the Hamaker constant of bacteria-water-surface is smaller than that of bacteria-air-surface, the changes in distance effects are more dominant on the van der Waals forces for this system studied in this work (roughness increases from ∼(2–40 nm) to ∼(50–390 nm) while the Hamaker constant increases from 3.29 × 10^–20^ J to 7.64 × 10^–20^ J upon changing medium from water to air based on the Lifshitz theory). Flattening and spreading of bacteria on a surface is governed by the interplay between the adhesive force and the elastic deformation force. Previous studies reported that Young’s modulus of a bacterial wall is in the order of 0.01 to 0.1 GPa for many bacteria.^92^ While the typical force needed to detach a bacterium from a surface depends on the nature of the surface and the bacterium, it usually ranges from 1 nN to 1 μN.^93^ Hence, assuming equidistribution of the adhesion force over the asperities underneath a bacterium and the validity of the Hertz contact theory with a cylinder on a flat geometry, one can roughly estimate that bacterial deformation is on the order of ∼0.5 nm to ∼50 nm with the upper and lower limits of moduli and forces reported above. SEM micrographs in Figure 3 seem to be more consistent with larger deformation values.

An order of magnitude analysis of the Laplace pressure yields an upper and lower limit of 2.6 and 0.4 MPa, respectively, assuming that rms roughness is equal to the radius of curvature with spherical enclosures. The multiplication of this pressure with the projected bacterial area of contact (2 μm × 1 μm) results in a pressure force of 0.7 to 5 μN, which is larger than the forces required to detach a single bacterium from a surface.^93^

## Conclusions

Overall, in this study, we investigate the effect of substrate surface roughness on the adhesion behavior of bacteria to model hydrophobic surfaces (methylated quartz) of systematically varying roughness from ∼2 nm to ∼390 nm but the same surface chemistry and (methyl group) coverage. The combination of the surface roughness and methylation chemistry resulted in hydrophobic and superhydrophobic behavior. The variations in surface roughness could account for 75-fold variation in the number of adhering bacteria, indicating the importance of surface topography and the presence of interstitial air for such processes. For hydrophobic surfaces, a strong correlation between roughness ratio and bacterial adhesion was obtained while autocorrelation length (related to the interasperity spacing) was not found to be correlated with bacterial adhesion. Both increased effective surface area with increasing roughness and decreased activation energy with increased surface roughness were concluded to enhance the extent of bacterial adhesion. For the cases of superhydrophobic surfaces, the combination of factors included (i) the surpassing of Laplace pressure force of interstitial air over bacterial adhesive force, (ii) the reduced effective substrate area for bacteria wall due to air gaps to have direct contact, and (iii) the reduction of the attractive van der Waals force that holds adhering bacteria on the substrate (the energy barrier of bacterial desorption/removal). These findings were validated for Gram-negative *Salmonella typhimurium* LT2 and *Escherichia coli* O157:H7 as well as Gram-positive *Listeria innocua*. Overall, this study brings about new insights into bacterial adhesion in the context of surface roughness. We anticipate that such knowledge is important for the design of engineering surfaces, coatings, devices, components, and systems as well as the understanding bacterial contamination scenarios emerging in various fields.

## Data Availability

The anonymized data that supports the findings of this study are available from the corresponding author upon reasonable request.

## Supporting Information Available

ATR-FTIR and XPS spectra for quartz, AFM micrograph for bacterial cells, larger SEM images, detailed explanation for the SEI simulation, and further data analysis (PDF)
