# Supplementary material for: Influence of Surface Roughness, Nanostructure, and Wetting on Bacterial Adhesion
Source: Langmuir. 2023 Apr 4;39(15):5426–39. doi: 10.1021/acs.langmuir.3c00091 (PMC10848269; doi:10.1021/acs.langmuir.3c00091)
Supplement: Supplementary file 1 — la3c00091_si_001.pdf [file la3c00091_si_001.pdf]

Supporting information

Influence of surface roughness, nanostructure, and wetting on bacterial adhesion

Minchen Mu [a,1], Shuhao Liu [a,1], William DeFlorio [a], Li Hao [b], Xunhao Wang [c], Karla Solis Salazar [d], Matthew Taylor [d], Alejandro Castillo [d], Luis Cisneros-Zevallos [e], Jun Kyun Oh [f,1], Younjin Min [c*], Mustafa Akbulut [a*]

[a] *Artie McFerrin Department of Chemical Engineering, Texas A&M University, College Station, TX 77843, USA*

[b] *School of Chemistry and Chemical Engineering, Zhongkai University of Agriculture and Engineering, Guangzhou, Guangdong, 510225, P.R. China*

[c] *Department of Chemical and Environmental Engineering, University of California, Riverside, CA 92521, USA*

[d] *Department of Food Science and Technology, Texas A&M University, College Station, TX 77843, USA*

[e] *Department of Horticultural Sciences, Texas A&M University, College Station, TX 77843, USA*

[f] *Department of Polymer Science and Engineering, Dankook University, 152 Jukjeon-ro, Suji-gu, Yongin-si, Gyeonggi-do 16890, Republic of Korea*

* Corresponding author.

E-mail address: makbulut@tamu.edu (M. Akbulut); Tel.: +1 979 847 8766; Fax: +1 979 845 6446.

E-mail address: younjinm@ucr.edu (Y. Min); Tel.: +1 951 827 6077; Fax: +1 951 827-5696.

[1] Both authors contributed equally to this work.

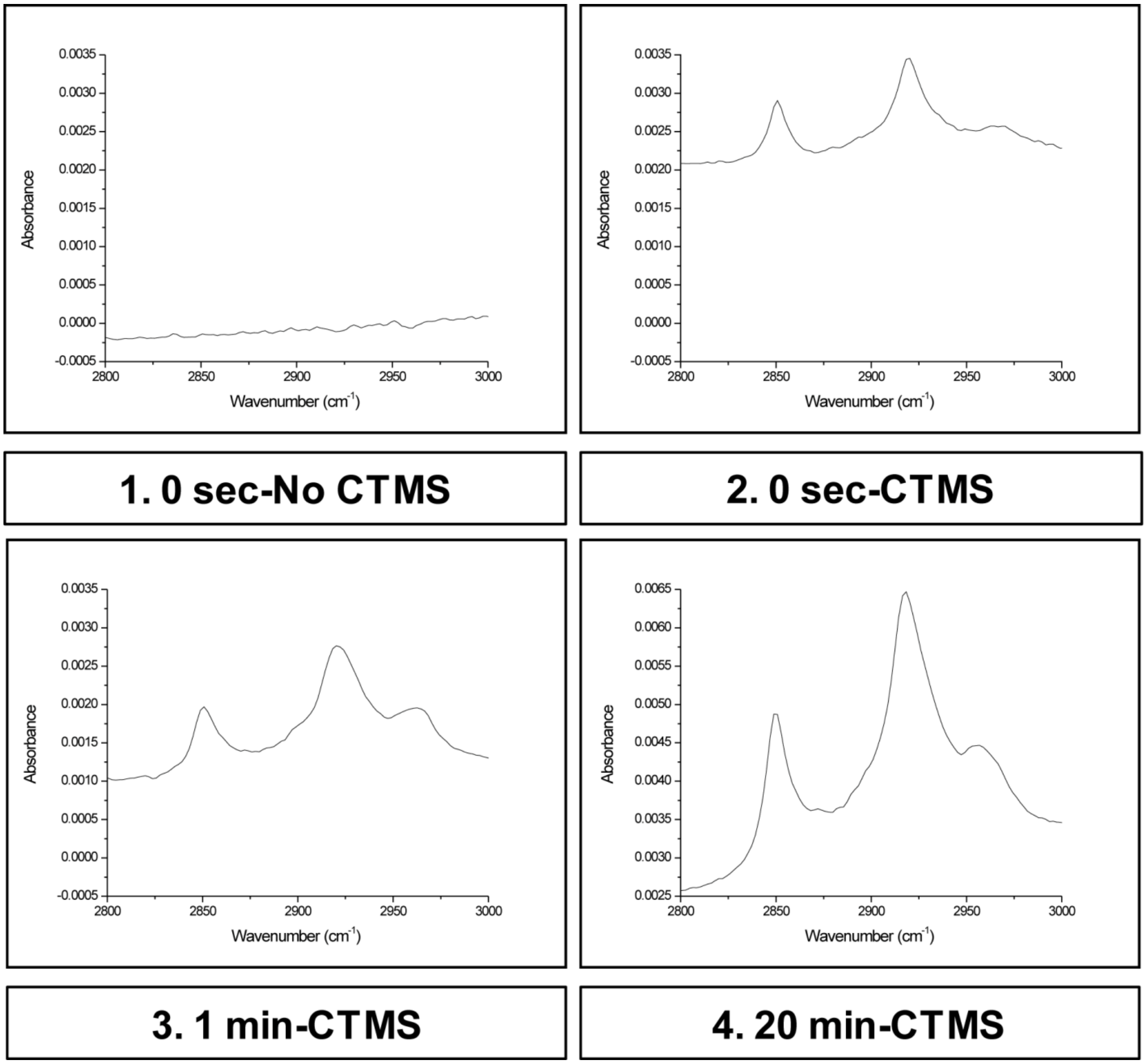

Fig. S1. ATR-FTIR spectra of TMCS, TMCS-functionalized quartz, TMCS-functionalized 1 min etched quartz, and TMCS-functionalized 20 min etched quartz. For the bare quartz, no –CH3 peak was found in the FTIR spectra. On the other hand, the FTIR spectra of the TMCS functionalized quartz had the same peak at 2850 cm^{-1} and 2950 cm^{-1} which corresponding to the $–CH_3$ stretch come from the TMCS modification and indicating the chemistry property of the TMCS modified surface are totally similar.

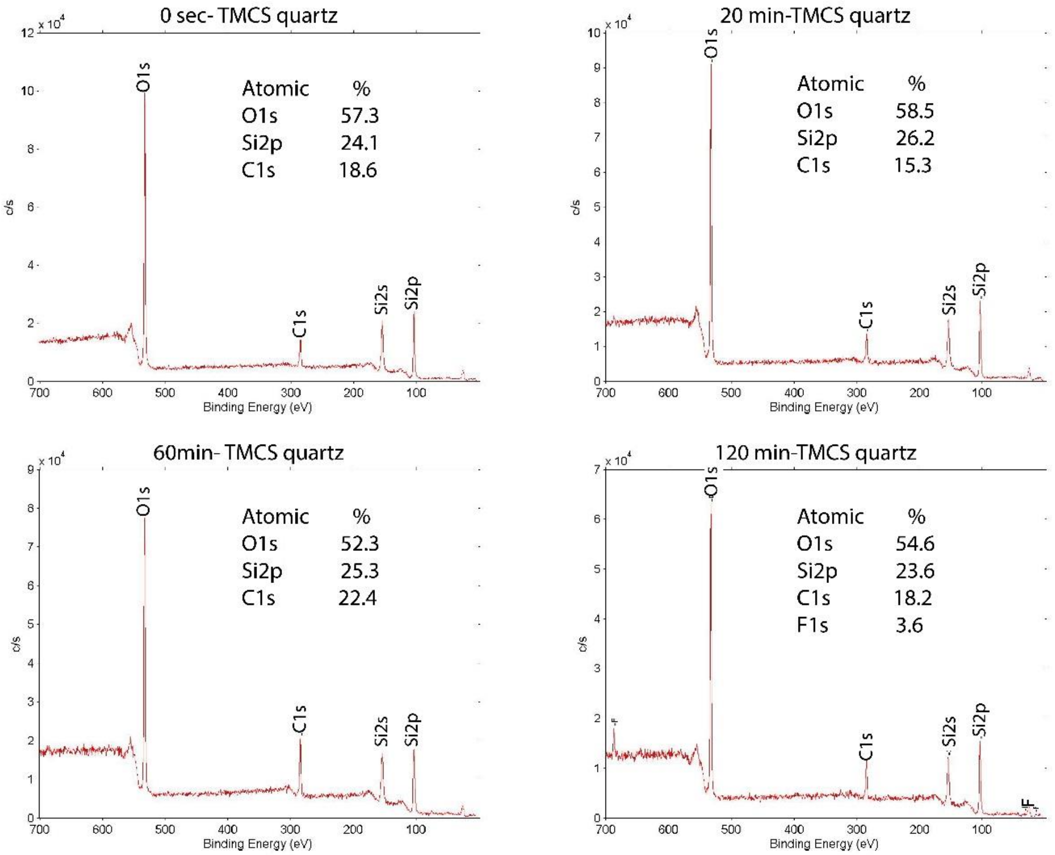

Fig. S2. The XPS spectra of TMCS-functionalized quartz, TMCS-functionalized 20 min etched quartz, TMCS-functionalized 60 mins etched quartz and TMCS functionalized 120 min etched quartz. The results indicating the coverage of the TMCS on the surface are similar for the quartz with varying time etching process.

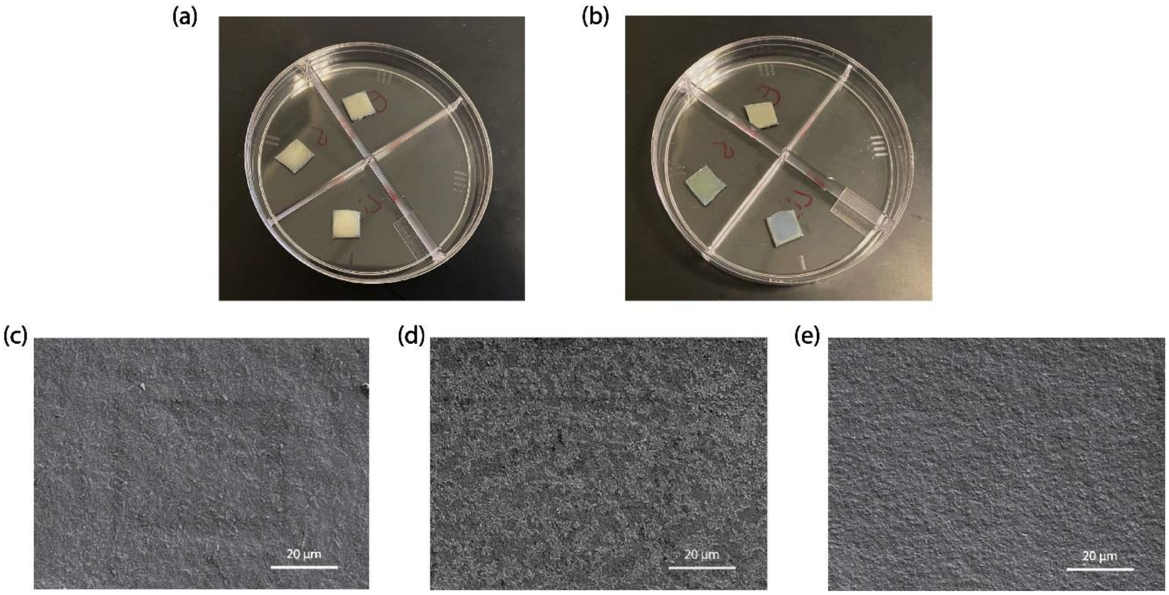

Fig. S3. (a) As-prepared bacterial lawns on silicon wafer. (b) Dehydrated bacterial lawns gained after 12h drying under room temperature, a complete layer of bacterial lawn was coated on the surface. (c-e) SEM images of bacterial lawns (*Salmonella, Listeria and E. coli*) at low magnification, which show that the silicon substrates are completely covered by bacterial cells.

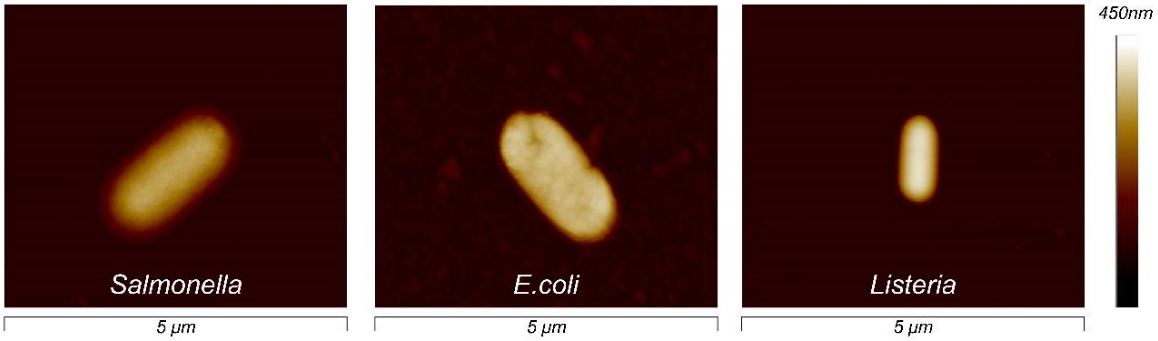

Fig. S4. The AFM of a single cell of *Salmonella*, *E. Coli*, and *Listeria*. Cells adhered on <100> silicon wafers.

Table. S1. Summary of the experimental conditions

**Surface chemistry**	Methylated quartz
**Preparation method**	Oxygen plasma etching
**Surface roughness**	2 nm - 390 nm
**Water contact angle of surface**	95.5° - 152.8°
**Bacteria type**	*Salmonella*, *Listeria*, *E. coli*
**Concentration of bacterial suspension**	8.8 ± 0.2 log10 CFU/mL (*S*),<br>9.1 ± 0.2 log10 CFU/mL (*L*),<br>8.6 ± 0.3 log10 CFU/mL (*E*)
**Volume of bacterial suspension for each sample**	9 mL
**Inoculation time**	4 hours

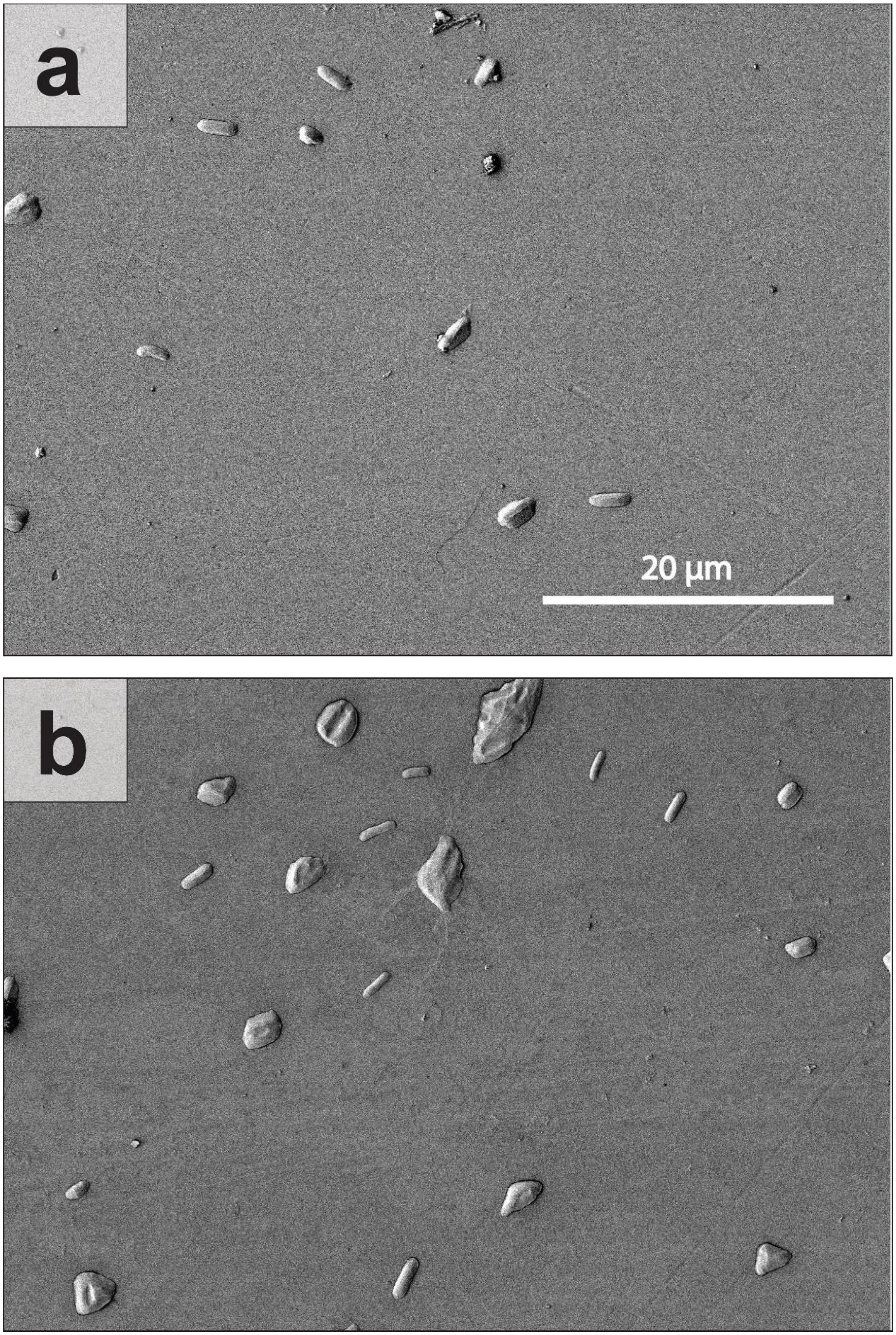
a
20 µm
b

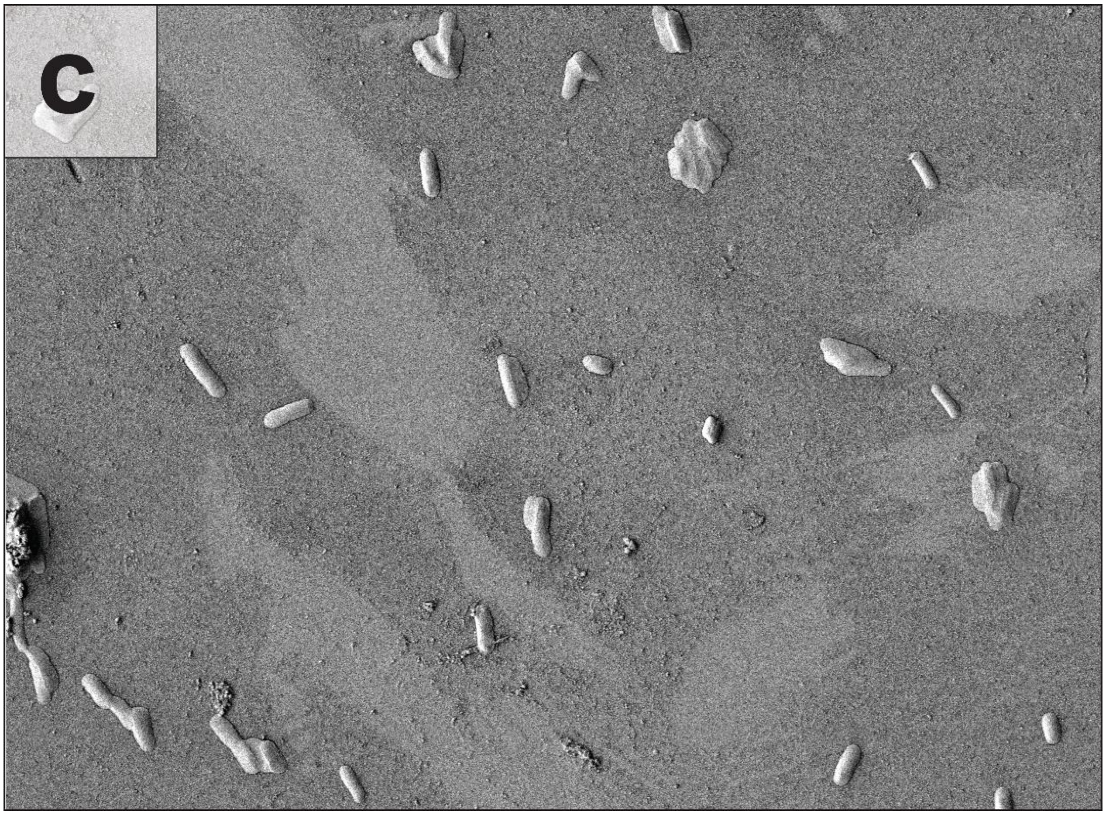
c

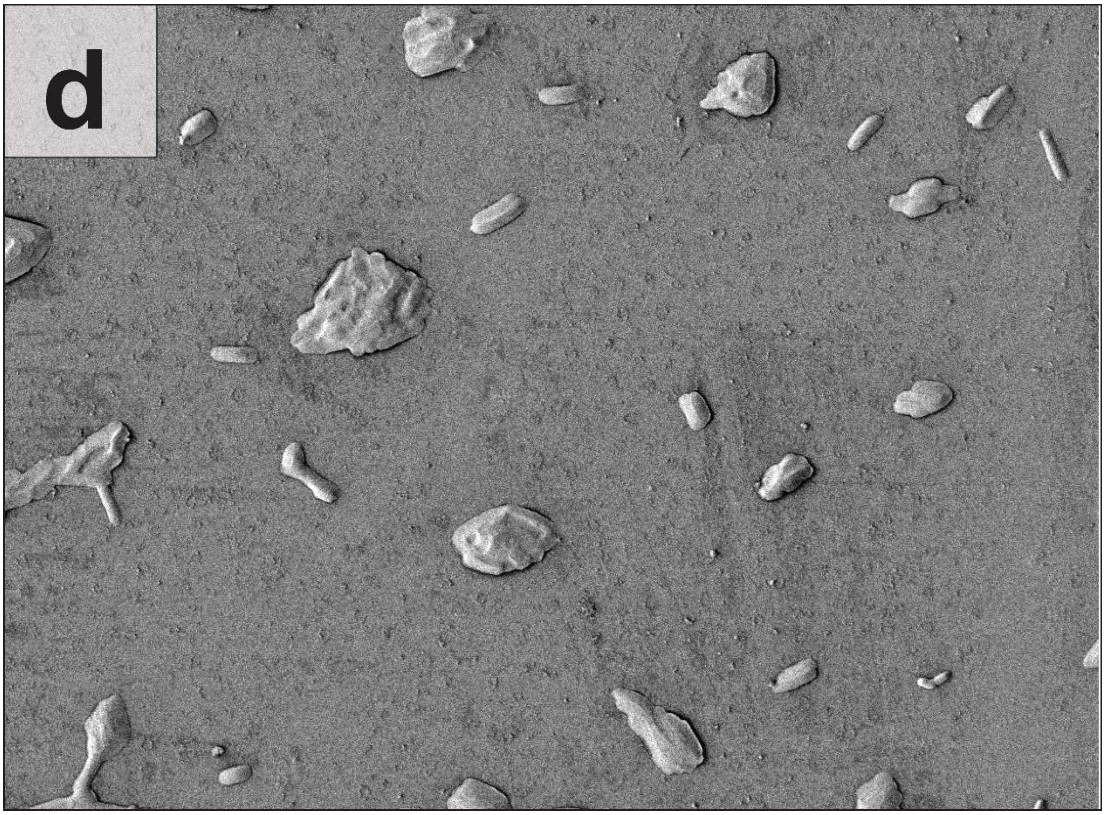
d

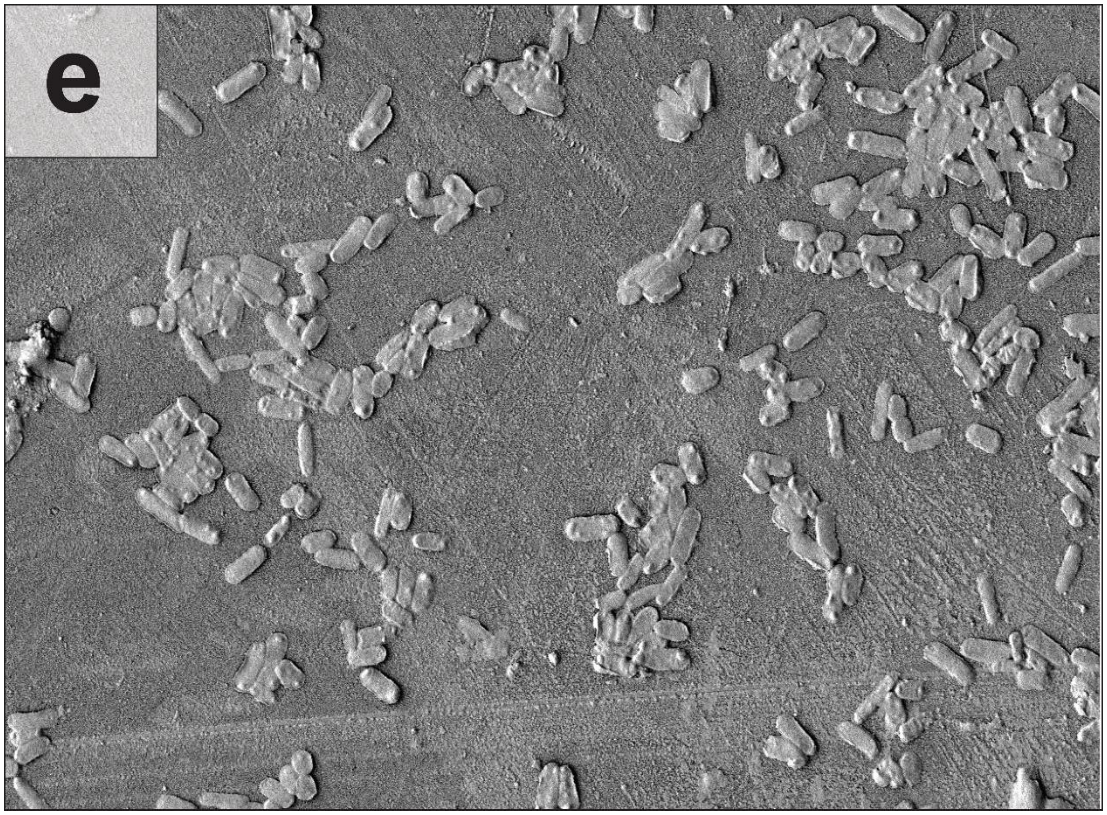
e

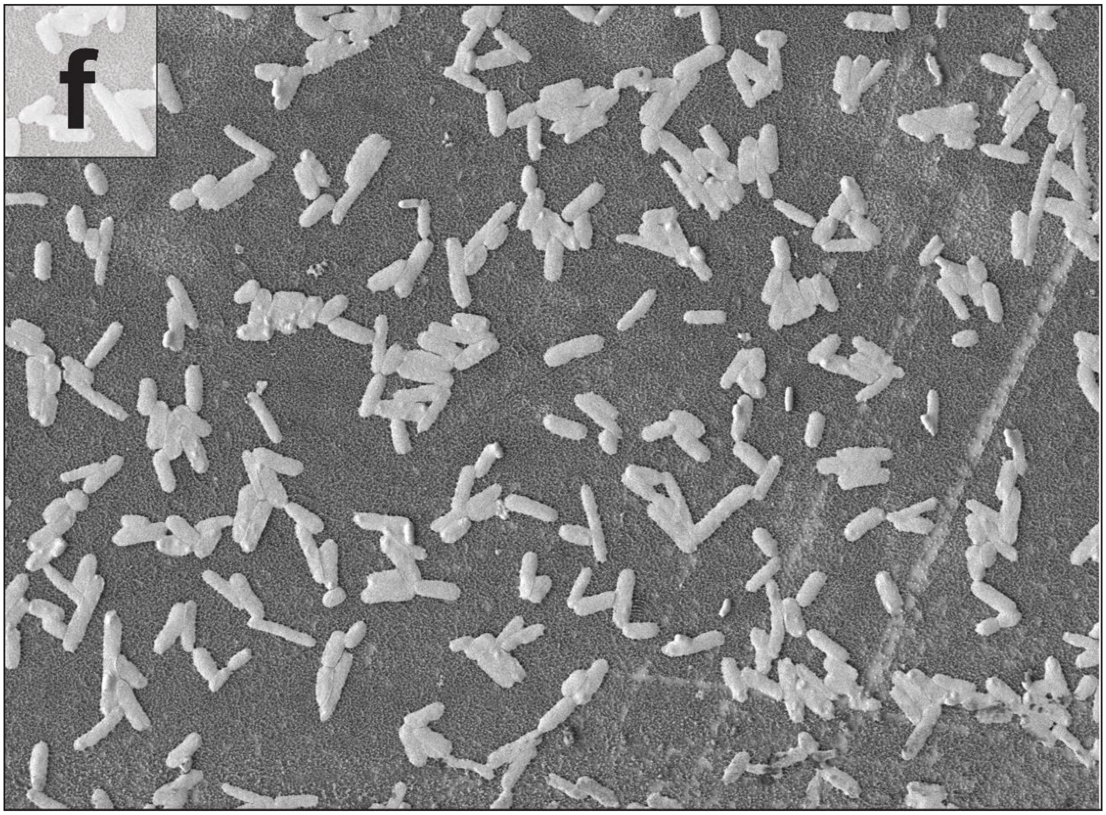
f

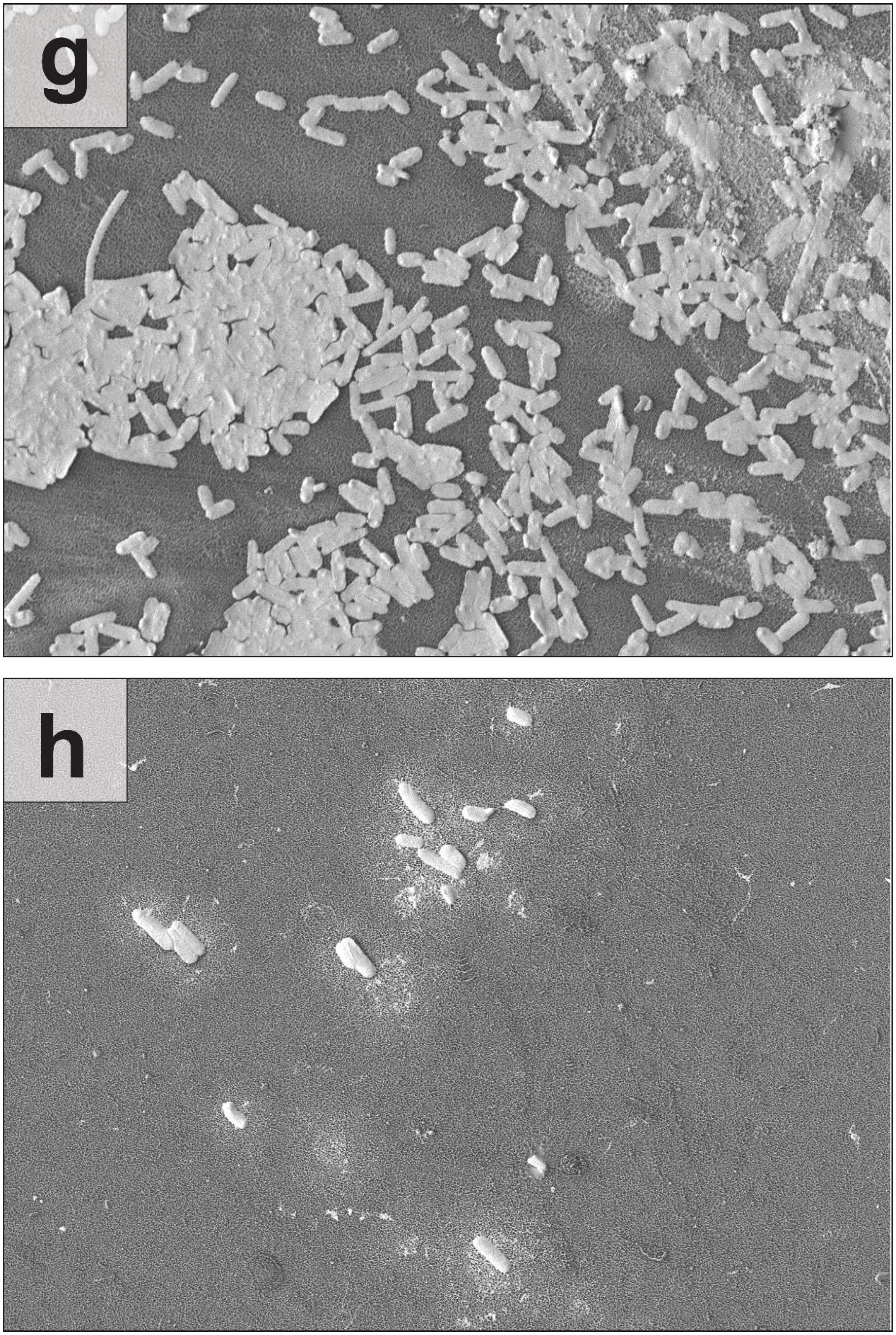
g
h

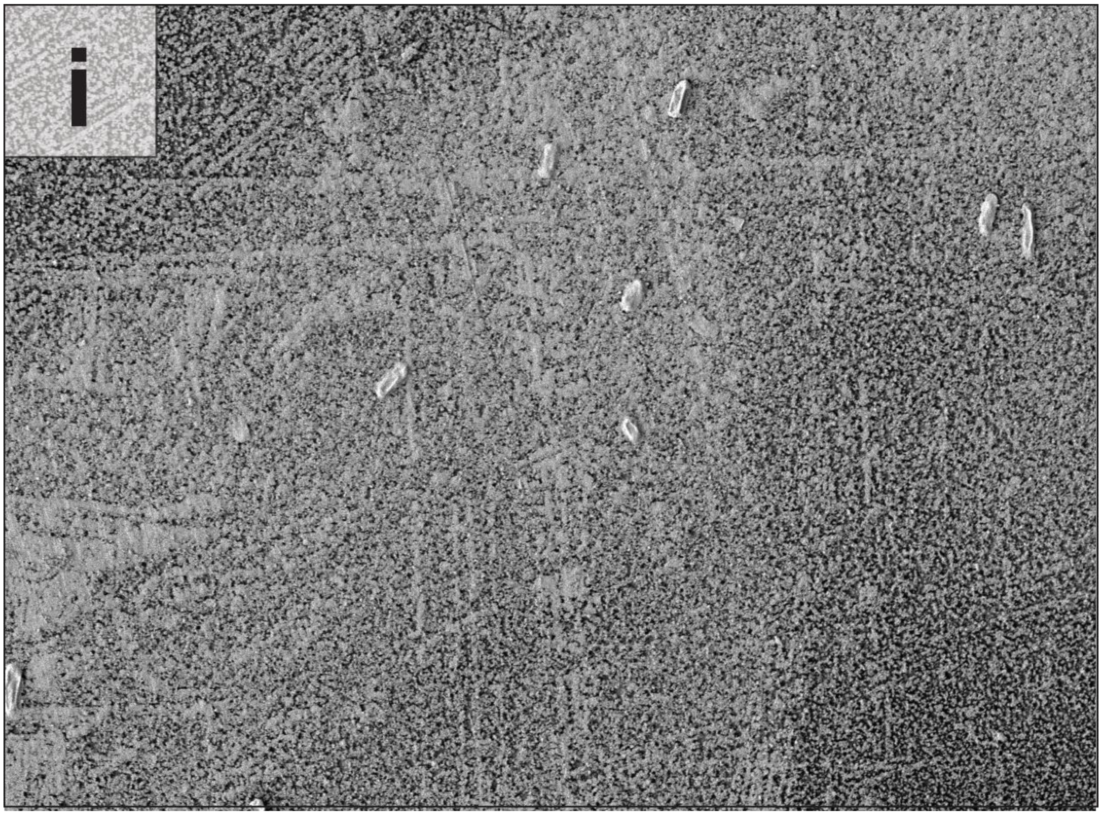
i

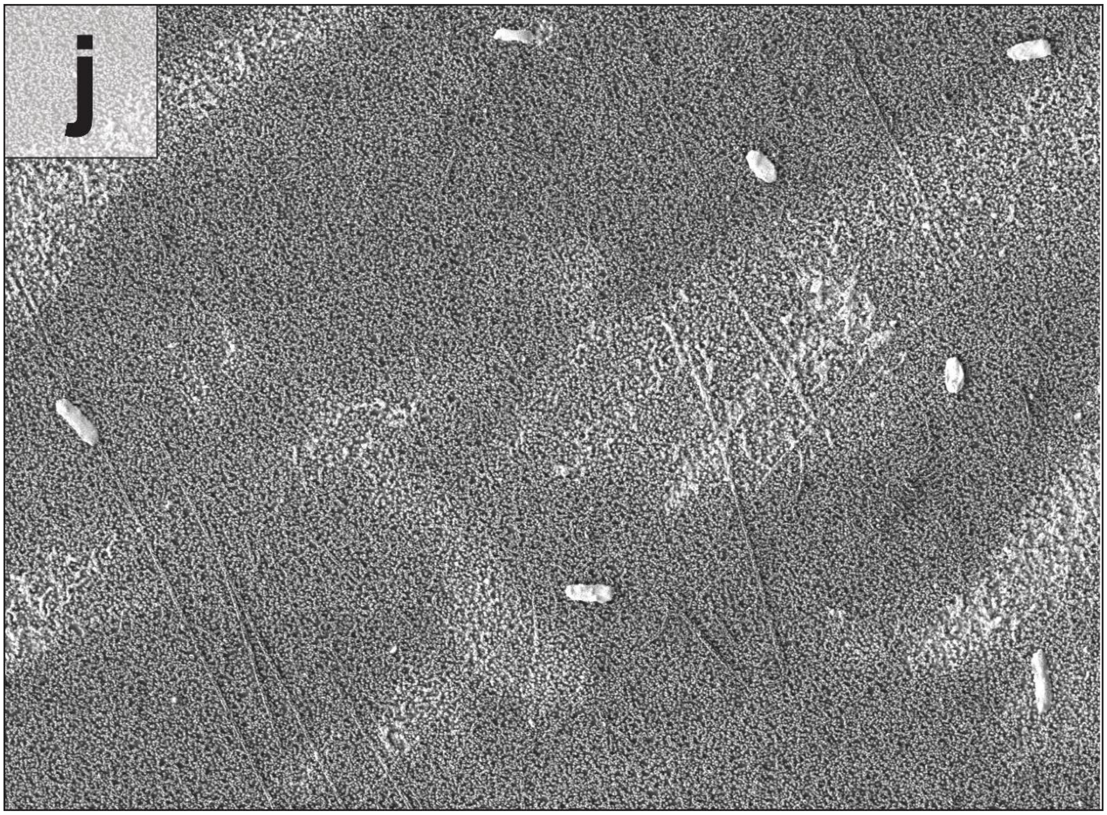
j

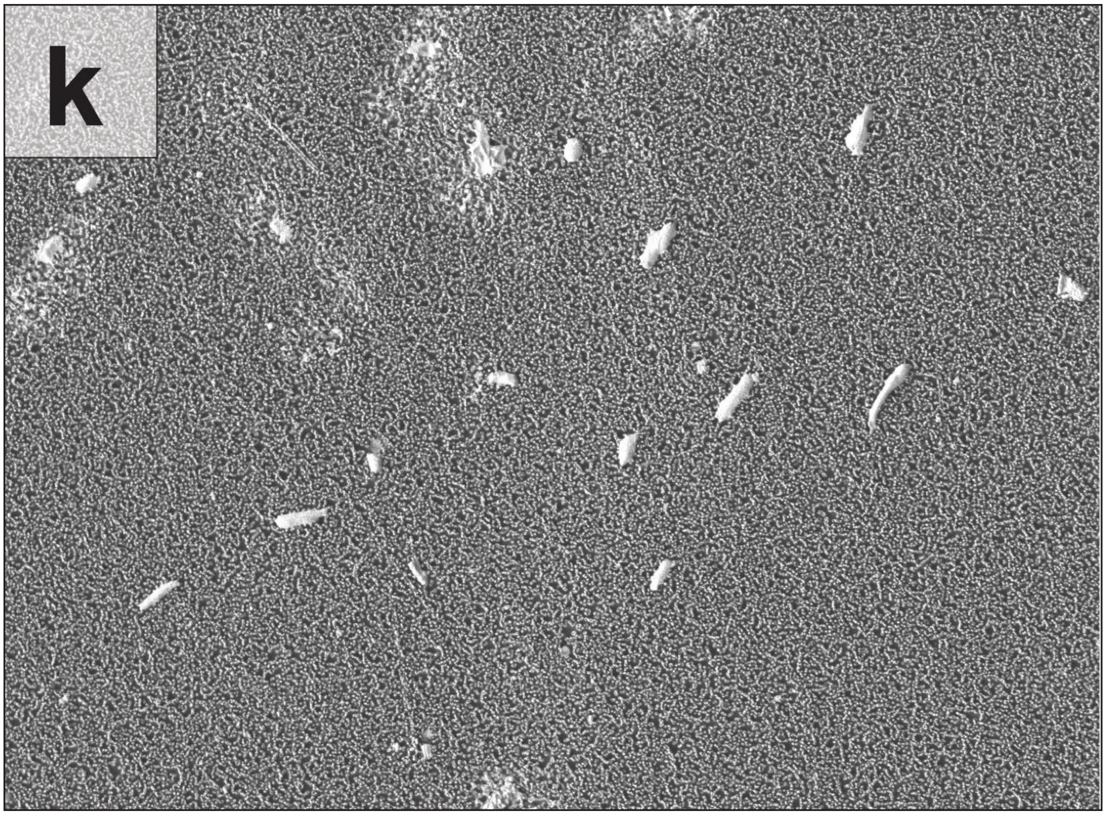
k

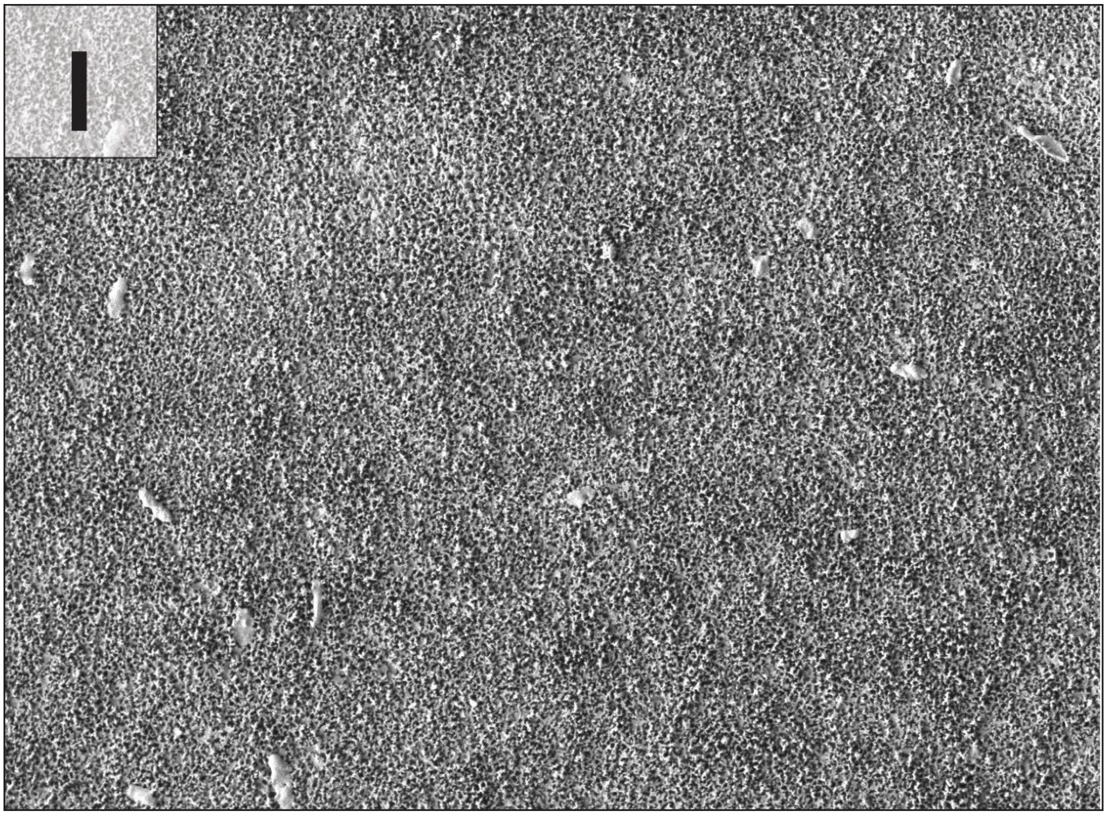
l

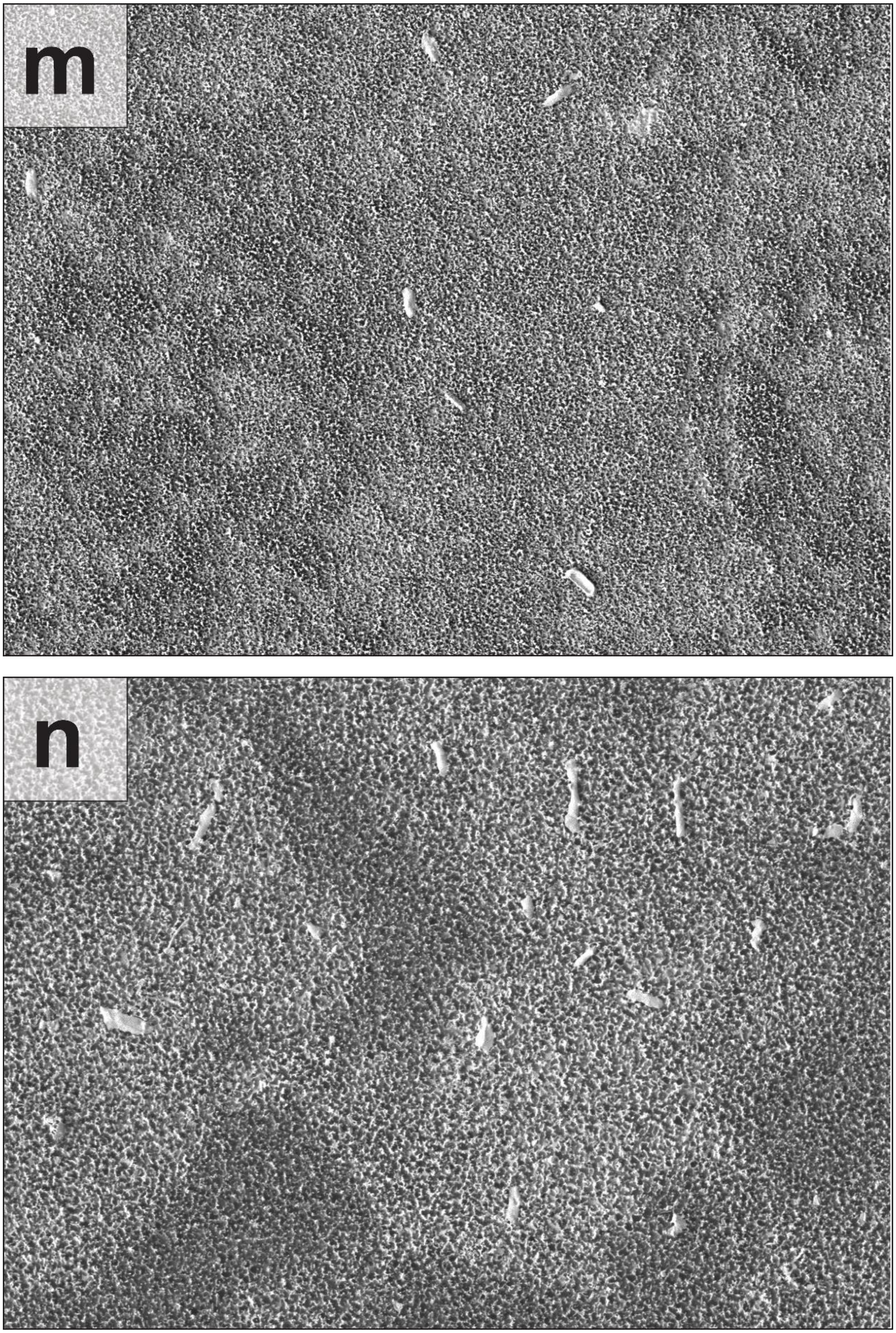
m
n

Fig. S5. (**a-g**) SEM micrographs (larger images) displaying bacterial adhesion trends on hydrophobically modified quartz surfaces with systematically varying surface roughness for *Salmonella*. Bacterial adhesion is significantly lower for superhydrophobic surfaces (samples **h–n**) compared to other samples ($p < 0.05$). The morphology of surfaces before bacterial inoculation is shown in Figure 2. Each micrograph has the same size of 45.6 μm × 60.7 μm.

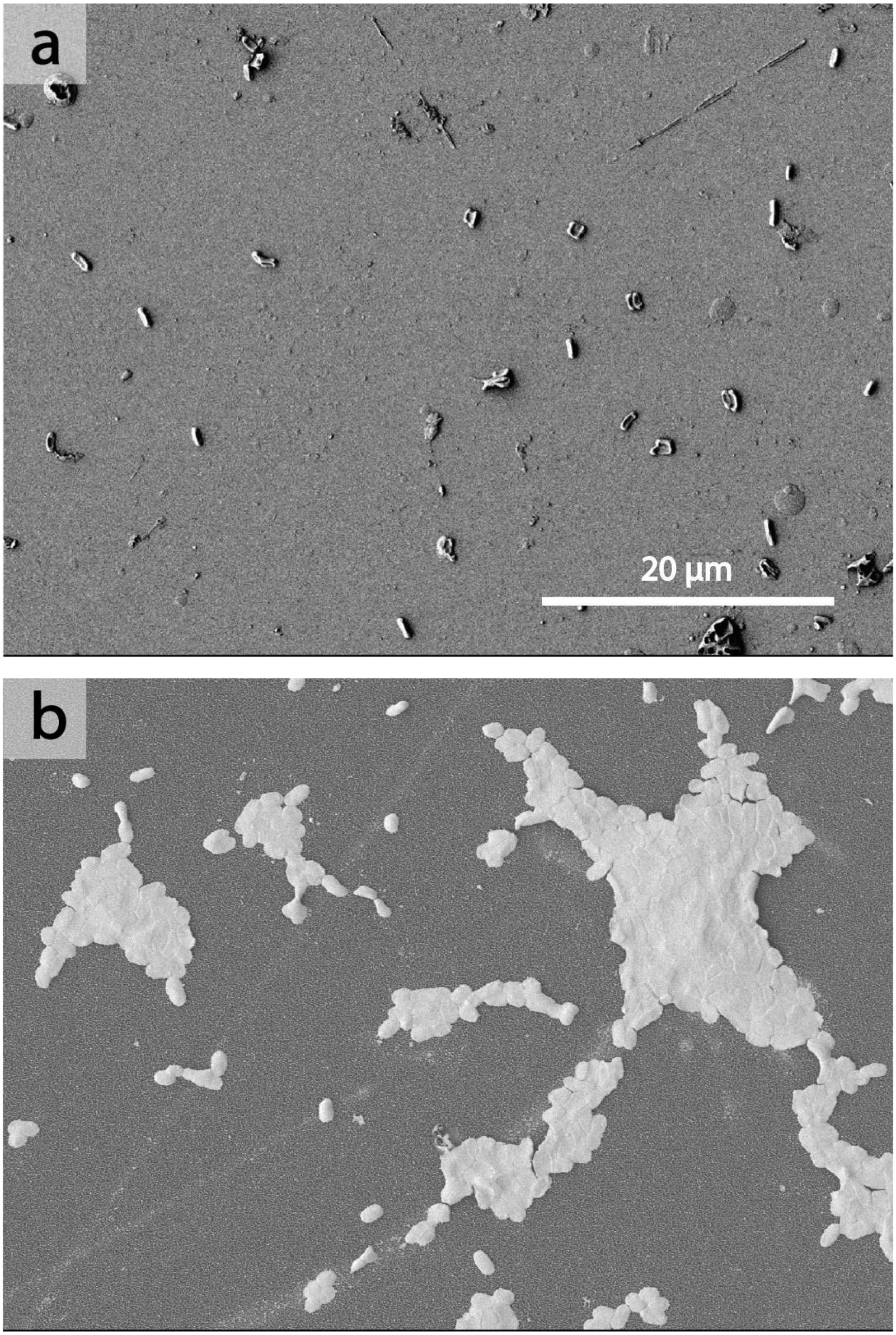
a
b
20 µm

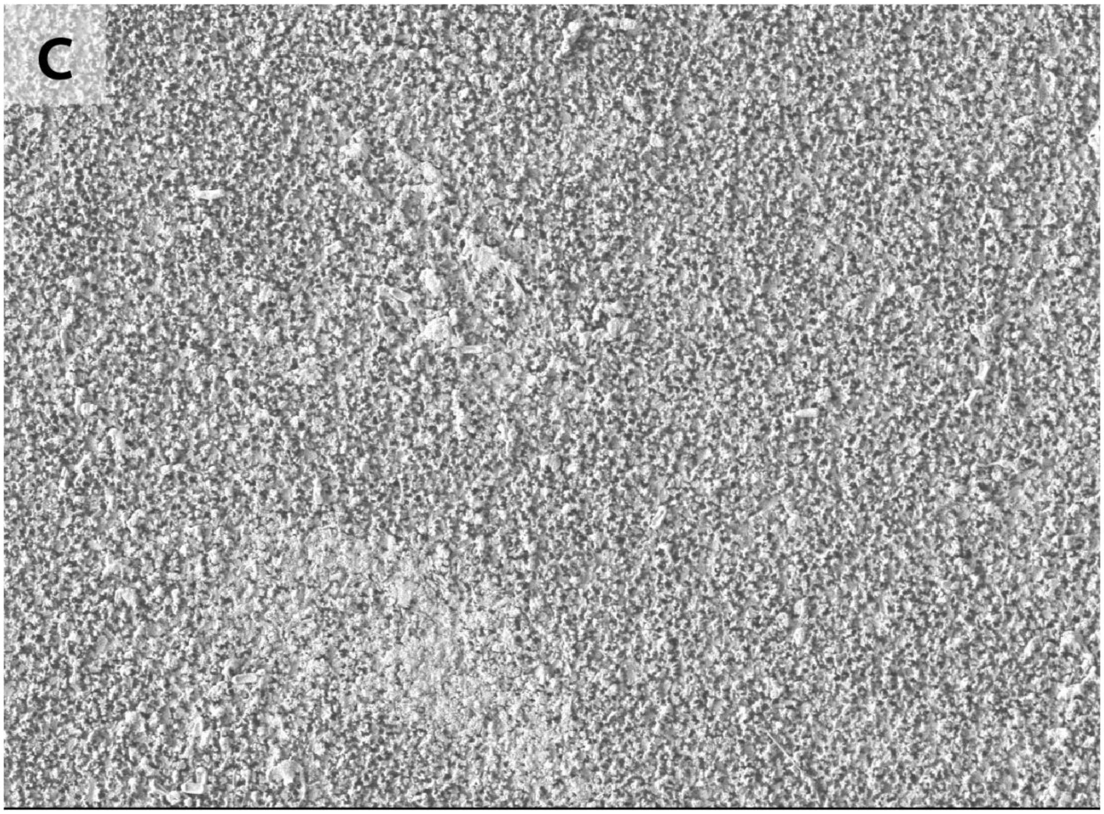
c

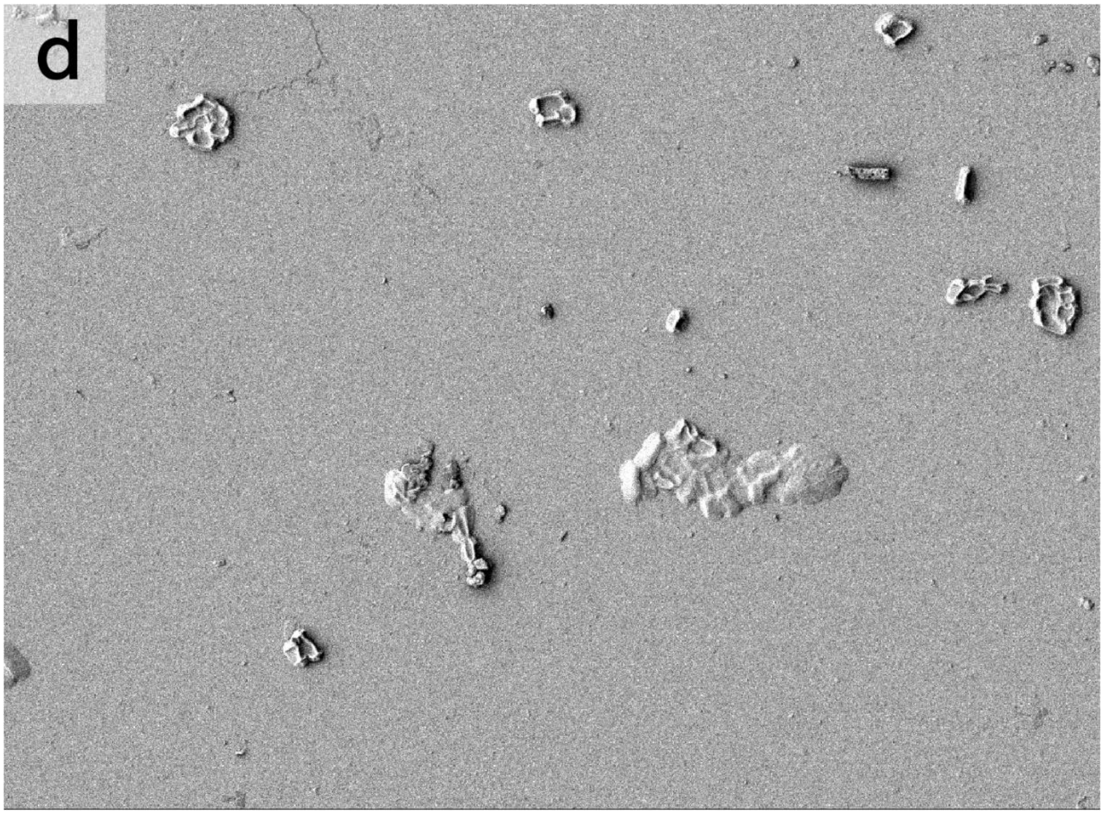
d

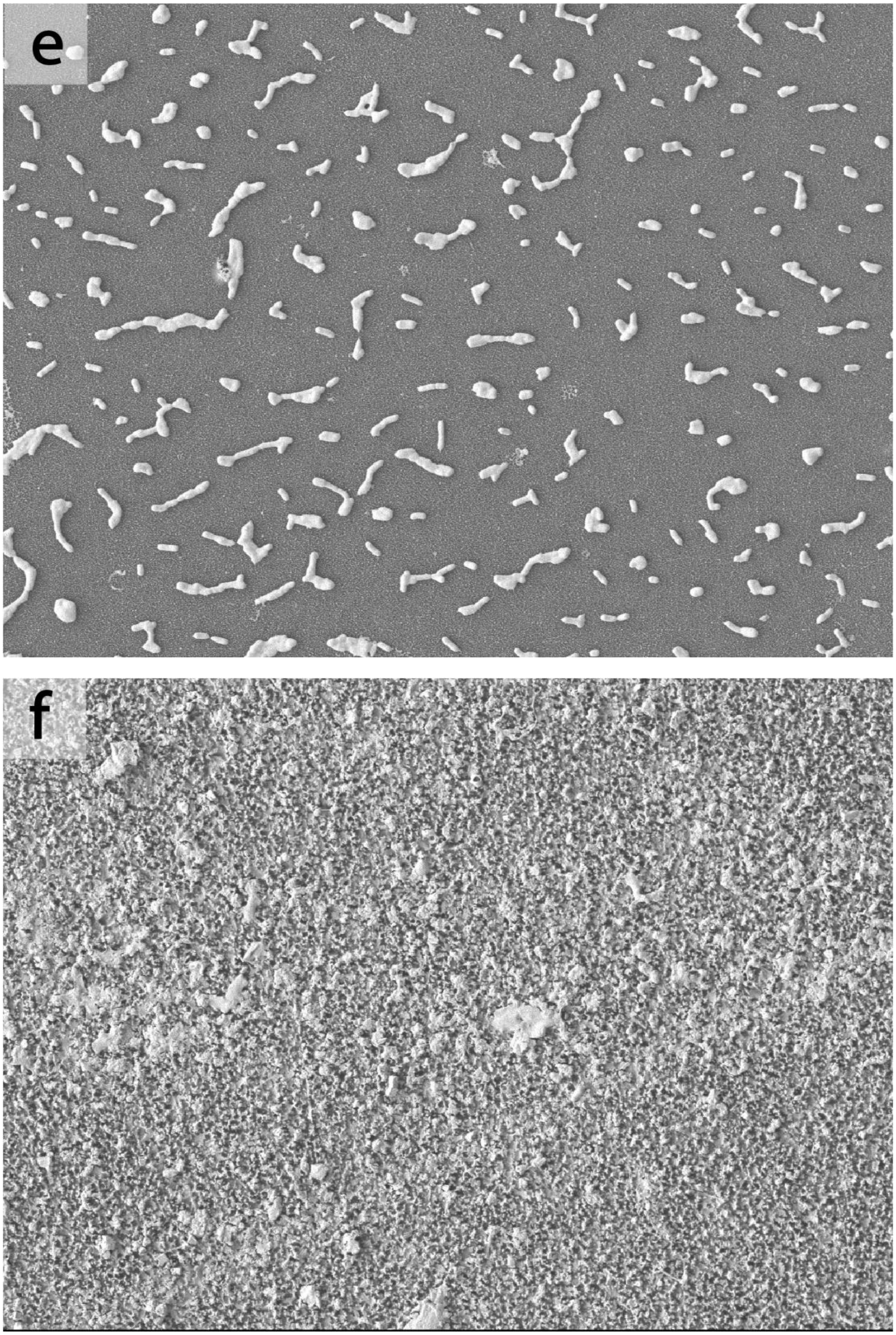
e
f

Fig. S6. SEM micrographs (larger images) displaying bacterial adhesion trends on three typical substrates, relatively smooth surface (hydrophobic), moderately rough surface (hydrophobic) and rough surface (superhydrophobic) for different bacteria. (**a-c**) *Listeria*. (**d-f**) *E. coli.* Each micrograph has the same size of 45.6 μm × 60.7 μm.

Details of Surface Element Integration

The DLVO theory has been applied to predict the interaction energy between bacteria and surface to interpret the behavior of bacterial adhesion. While the original DLVO theory was developed for spherical particles, the DLVO based on SEI (surface element integration) enables the calculation for various shapes of molecules.[1–3] Here, we have developed a model consisted of a cylinder and a rough surface described by double-cosine function to simulate the bacteria cell and quartz surface with different roughness, respectively (Figure S5).

The interaction energy per unit area of the van der Waals and electrical double layer can be expressed by functions of separation distance h as:[4]

$$E_{VDW}(h) = -\frac{A}{12\pi d h^2}$$

$$E_{DL}(h) = 32\varepsilon_0\varepsilon_r\gamma_1\gamma_2 K\left(\frac{kT}{ve}\right)^2 \exp\left(-Kh\right)$$

where A is the Hamaker constant of this bacteria-water-quartz system obtained by:[4]

$$A_{bwq} = \sqrt{\left|(A_{bb} + A_{ww} - 2A_{bw})(A_{qq} + A_{ww} - 2A_{qw})\right|},$$

ε_0 , ε_r represent the permittivity of vacuum and water, γ is a parameter related to zeta potential, K is the inverse of Debye length which calculated from the equivalent ionic concentration, k stands for Boltzmann constant, T represents temperature and v is the valence of solution.

To understand the influence of roughness(rms), we modeled the rough surface by the wave of double-cosine function: $y(x,z) = \delta cos(nx)\cos(nz)$. Various roughnesses, which significantly affect the separation distance between bacteria and the surface, can be investigated by changing the value of δ and n, and further confirmed by the mathematic formula of *RMS*. We assumed the bacteria cell is lied down and parallel to the surface. Hence, the total interaction energy profile could be obtained by doing surface element integration of the cylinder as:

$$W_{Total}(h) = W_{VDW}(h) + W_{DL}(h)$$

$$h = D - asin\theta + \delta - \delta cos\theta(nacos\theta)\cos(nz)$$

$$W_{VDW}(h) = -\frac{Aa}{12\pi}\int_{-L}^{L}\int_{0}^{\pi} sin\theta \left[\frac{1}{(D - asin\theta + \delta - \delta cos\theta(nacos\theta)\cos(nz))^2} + \frac{1}{(D + asin\theta + \delta - \delta cos\theta(nacos\theta)\cos(nz))^2} \right] d\theta dz$$

$$W_{DL}(h) =$$

$$32\varepsilon_0\varepsilon_r\gamma_1\gamma_2 K\left(\frac{kT}{ve}\right)^2 \left[\begin{array}{c} \int_{-L}^{L}\int_{0}^{\pi} sin\theta \exp\left(-K(D - asin\theta + \delta - \delta cos\theta(nacos\theta)\cos(nz))\right) d\theta dz \\ + \int_{-L}^{L}\int_{0}^{\pi} sin\theta \exp\left(-K(D + asin\theta + \delta - \delta cos\theta(nacos\theta)\cos(nz))\right) d\theta dz \end{array} \right]$$

which mathematically shows that the roughness of surface affects the separation distance between bacteria and surface then further influence the total interaction energy.

Figure S6 shows the total interaction energy (scaled to kT) between a *salmonella* cell and the quartz surface within the hydrophobic region (*RMS* < 40 nm). With the increase of separation distance from 0.1 nm to 200 nm, the interaction energy increases, then decreases and trends to zero. The positive peaks appear at 1–2 nm with a slight difference on the location according to different surface roughness, regarded as the interaction energy barrier for the bacterial adhesion due to the larger magnitude of double layer repulsive forces comparing with the van der Waal attractive forces. Figure 3 shows the dependence of activation energy (E_a) on the surface roughness (*RMS*). The results indicate that it is easier for bacteria to overcome the interaction energy barrier and adhere to the rougher surface, which is in agreement with the experimental behaviors of the bacterial adhesion on the hydrophobic surfaces. The activation energy is negative linear to the surface roughness, varying by a factor about 3. Similar simulation method was used to predict the behavior of *Listeria* and *E. Coli.* While the peak value of activation energy varies due to various characteristic length and width as well as the zeta potential of different bacteria, the tendency that the activation energy decreases as surface roughness increases keeps consistent.

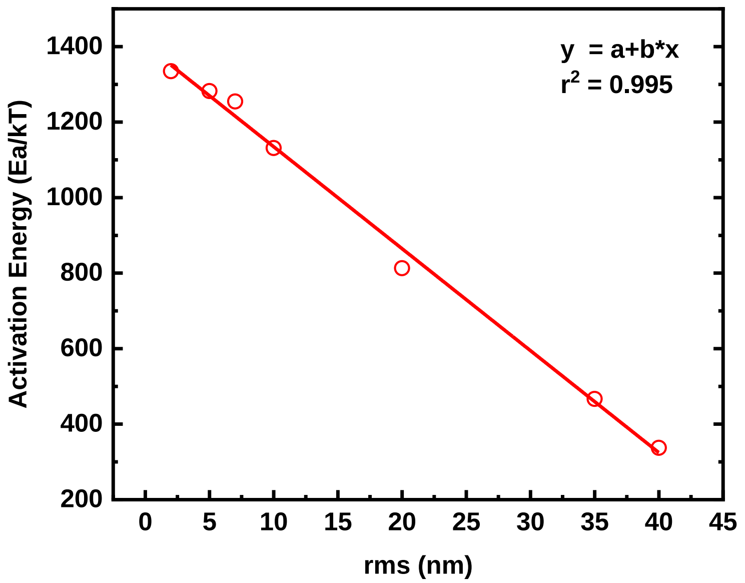

Fig. S7. Activation energy of bacterial adhesion for different surface roughnesses for *Salmonella.*

Table S2. The Statistical analysis ANOVA with Tukey's post-hoc test of the density of bacterial adhesion trends of *Salmonella, Listeria*, and *E. coli* on methylated quartz surfaces (#/mm^2) with varying Cassie fractions ($p<0.05$). Levels not designated by same letter are significantly different.

One-way Analysis of Roughness 0.79325 By Bacterial species

**Level**		**Mean** #/mm^2
Salmonella	A	23888.889
E. Coli	A	16203.704
Listeria	A	14537.037

One-way Analysis of Roughness 0.59231 By Bacterial species

**Level**		**Mean** #/mm^2
Salmonella	B	84444.444
E. Coli	B	65185.185
Listeria	B	48148.148

One-way Analysis of Roughness 0.22302 By Bacterial species

**Level**		**Mean** #/mm^2
Salmonella	C	145185.19
Listeria	C	92592.59
E. Coli	C	60740.74

One-way Analysis of Roughness 0.19699 By Bacterial species

**Level**		**Mean** #/mm^2
Listeria	A	7777.7778
E. Coli	A	3580.2469
Salmonella	A	2592.5926

References:

(1) Bhattacharjee, S.; Elimelech, M. Surface Element Integration: A Novel Technique for Evaluation of DLVO Interaction between a Particle and a Flat Plate. *Journal of colloid and interface science* **1997**, *193* (2), 273–285.

(2) Rajupet, S. DLVO Interactions between Particles and Rough Surfaces: An Extended Surface Element Integration Method. *Langmuir* **2021**, *37* (45), 13208–13217.

(3) Hoek, E. M. V; Agarwal, G. K. Extended DLVO Interactions between Spherical Particles and Rough Surfaces. *Journal of Colloid and Interface science* **2006**, *298* (1), 50–58.

(4) Israelachvili, J. N. *Intermolecular and Surface Forces*, Third Edit.; Academic Press, 2011. https://doi.org/10.1016/B978-0-12-375182-9.10011-9.
